# Supplementary figures and images for: Kazrin promotes dynein/dynactin-dependent traffic from early to recycling endosomes
Source: eLife. 2023 Apr 25;12:e83793. doi: 10.7554/eLife.83793 (PMC10181827; doi:10.7554/eLife.83793)

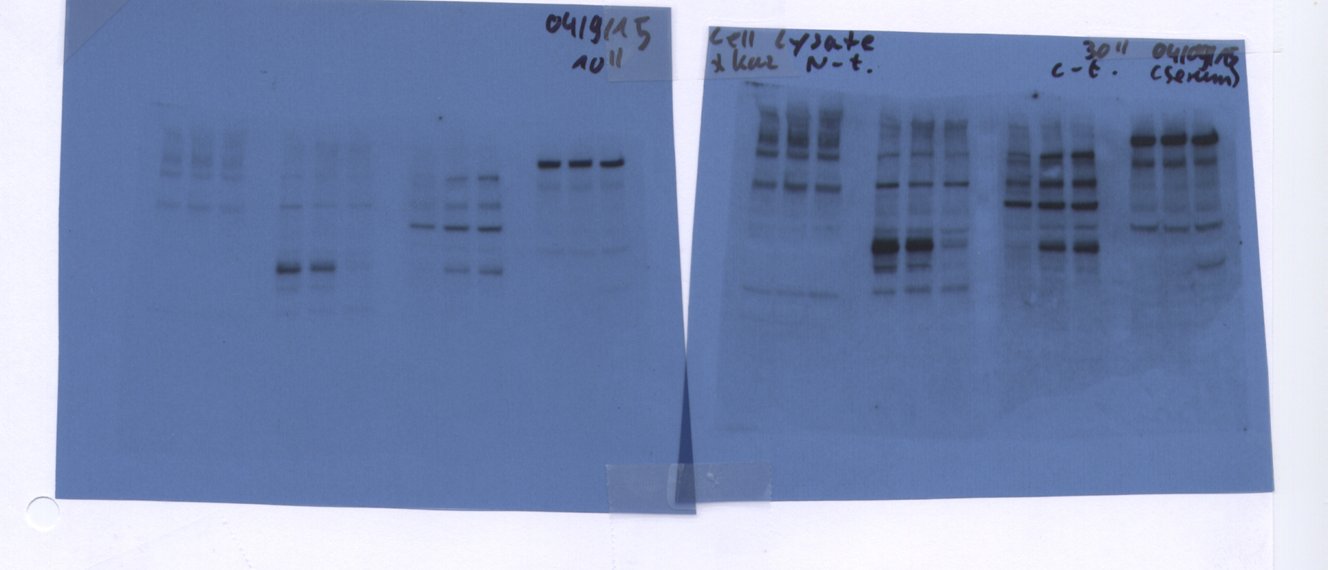

Supplement: Figure 1—figure supplement 1—source data 1. [file elife-83793-fig1-figsupp1-data1.zip › FIGURE1-figure supplement1-source data1/Figure1-figure supplement1A.jpg]

## Cos 7 cells immunoblot

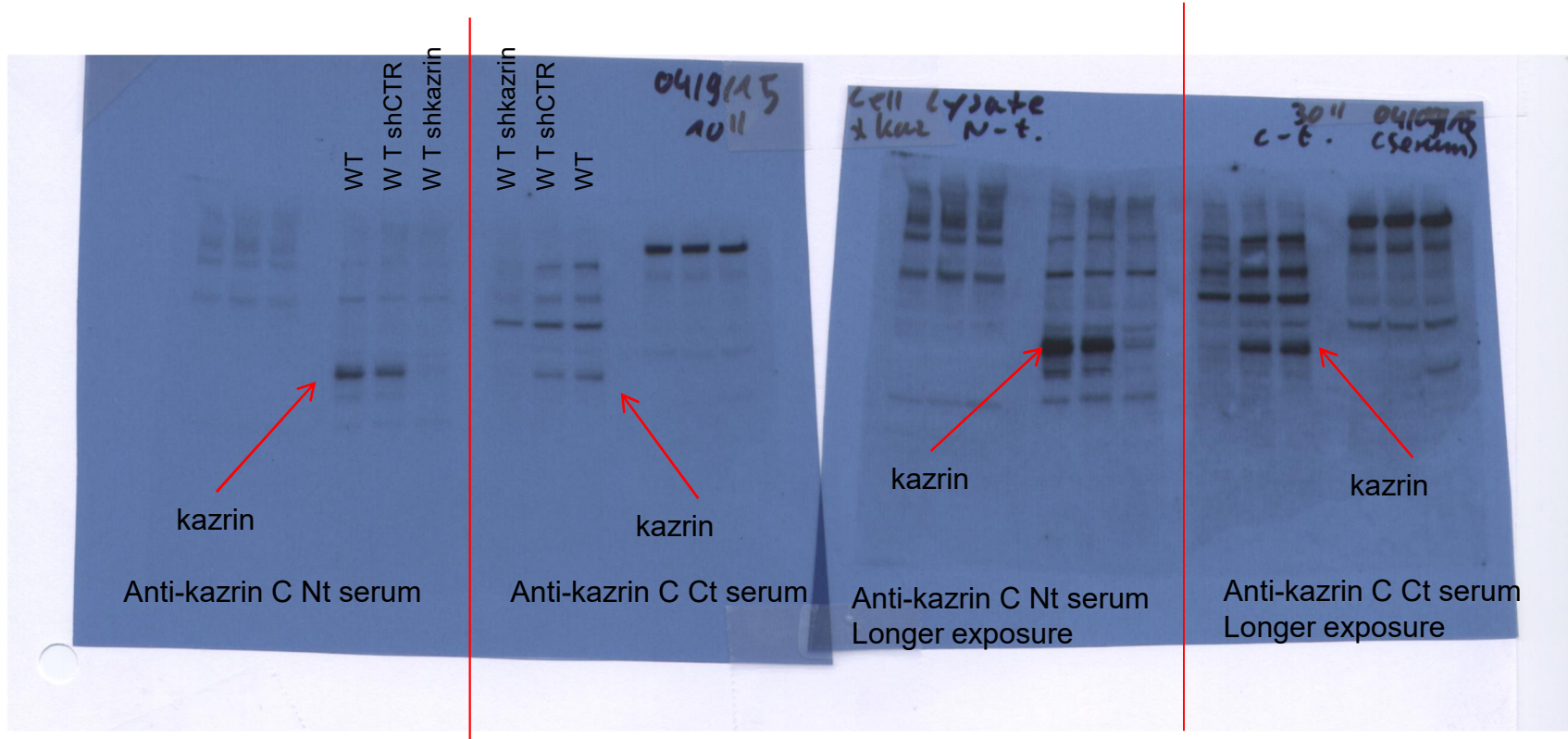

Supplement: Figure 1—figure supplement 1—source data 1. [file elife-83793-fig1-figsupp1-data1.zip › FIGURE1-figure supplement1-source data1/FIgure1-figure supplement1A.pdf]

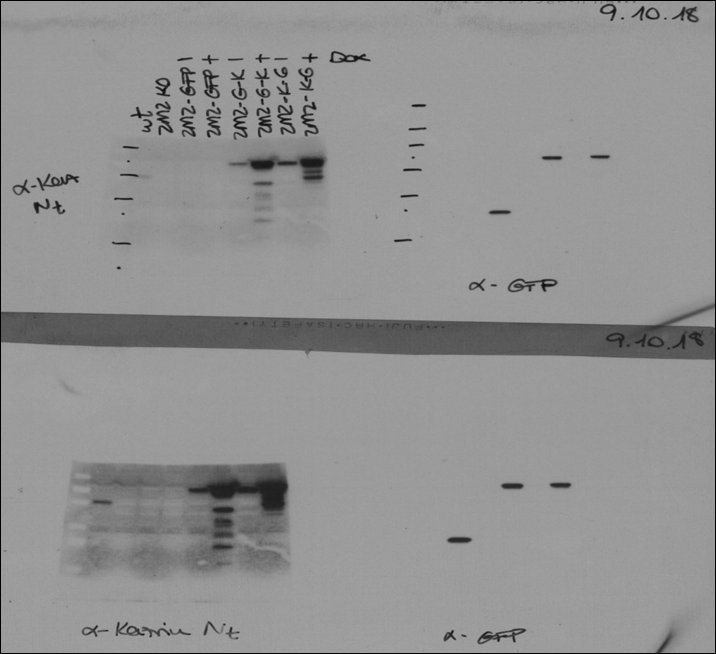

Supplement: Figure 1—figure supplement 2—source data 1. [file elife-83793-fig1-figsupp2-data1.zip › FIGURE1-figure supplement2-source data/Figure1-figure supplement2B.jpg]

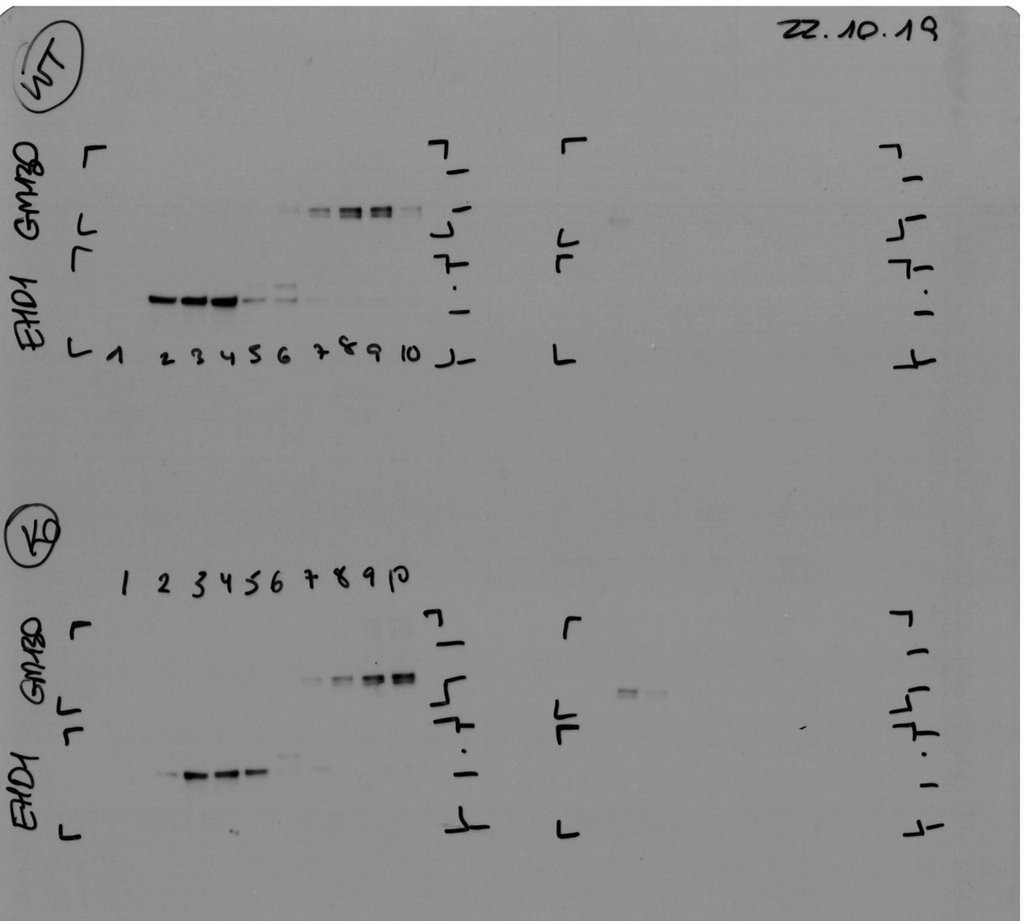

Supplement: Figure 3—source data 1. [file elife-83793-fig3-data1.zip › FIGURE3-source data/FIGURE3A/Figure 3A.EHD1 GM130.jpg]

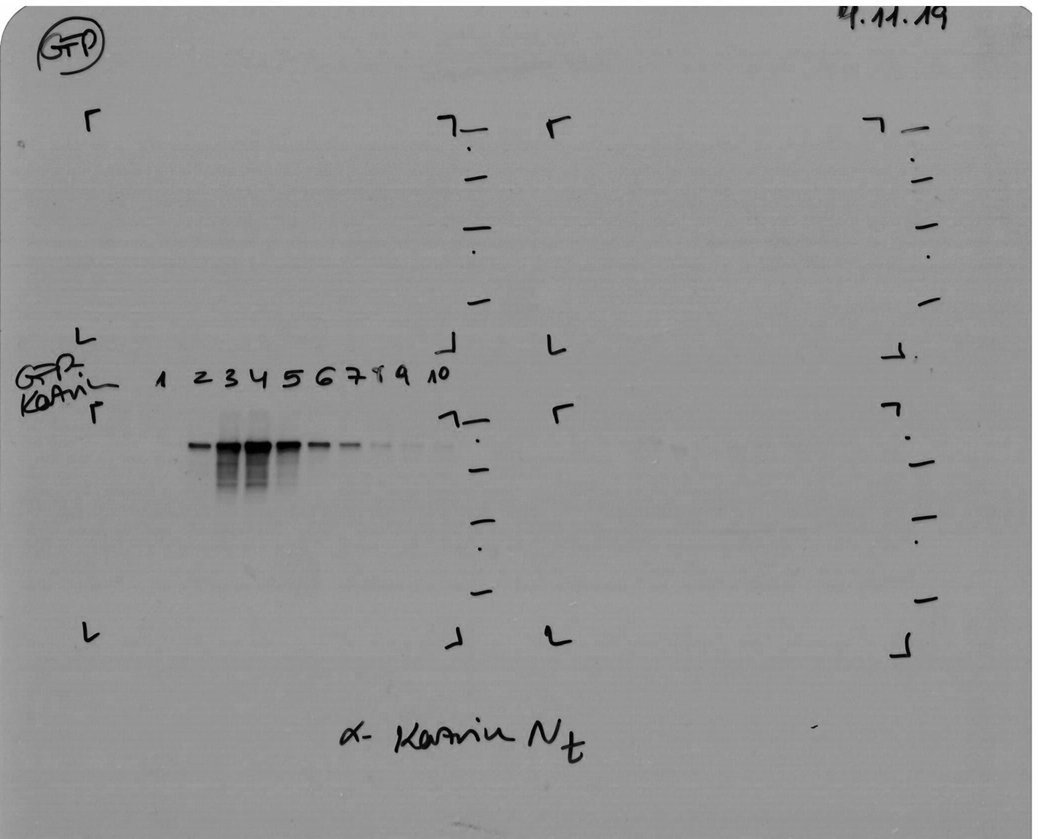

Supplement: Figure 3—source data 1. [file elife-83793-fig3-data1.zip › FIGURE3-source data/FIGURE3A/figure 3A.GFP-kazrin.jpg]

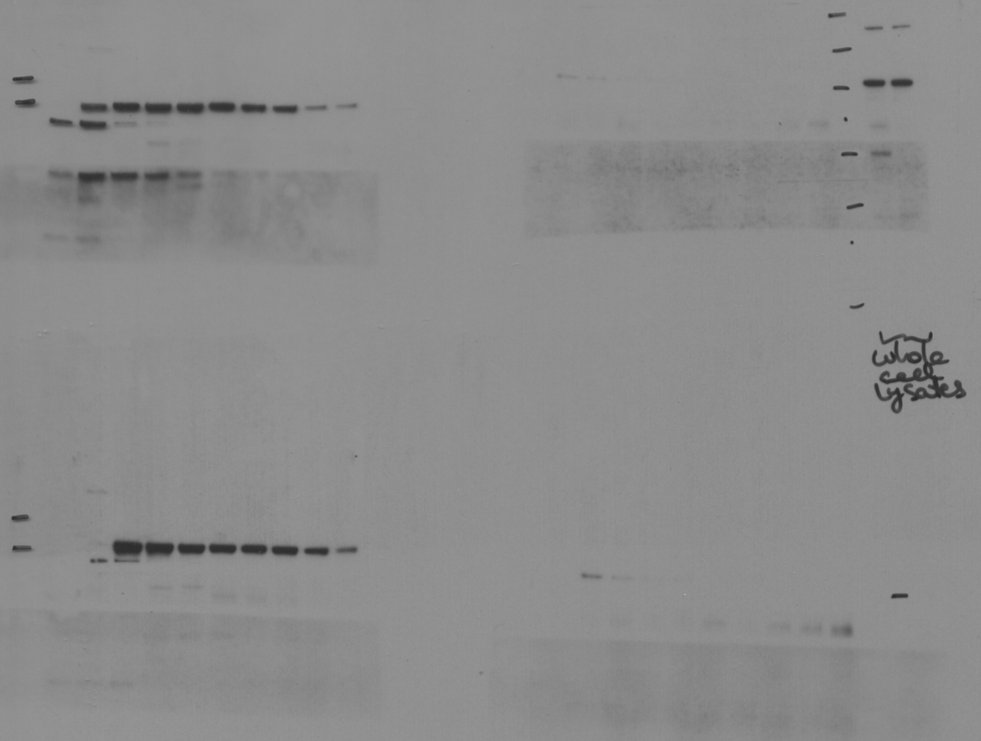

Supplement: Figure 3—source data 1. [file elife-83793-fig3-data1.zip › FIGURE3-source data/FIGURE3A/Figure 3A.kazrin gamma adaptin.jpg]

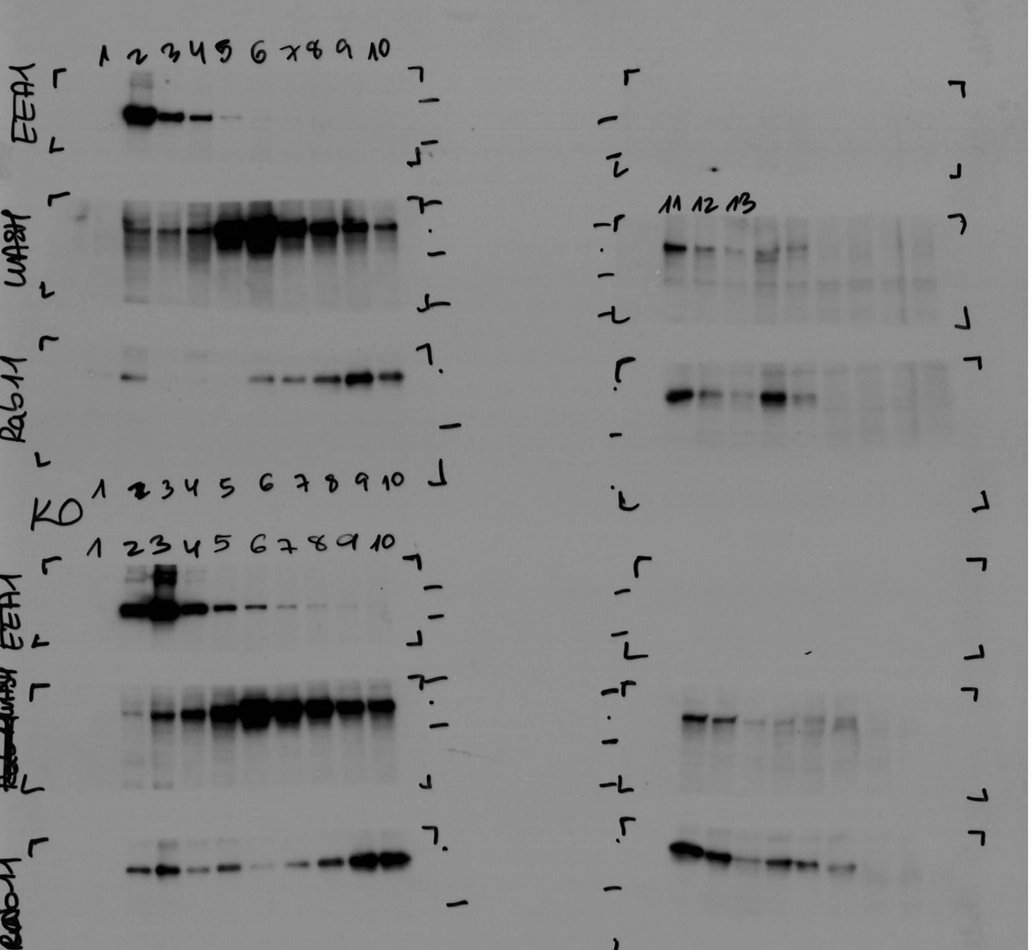

Supplement: Figure 3—source data 1. [file elife-83793-fig3-data1.zip › FIGURE3-source data/FIGURE3A/Figure3A.EEA1.Rab11.jpg]

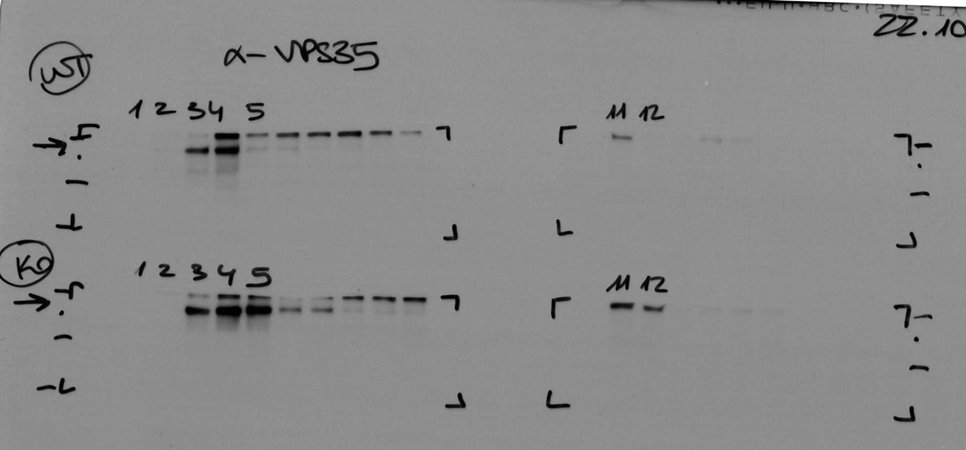

Supplement: Figure 3—source data 1. [file elife-83793-fig3-data1.zip › FIGURE3-source data/FIGURE3A/Figure3A.vps35.jpg]

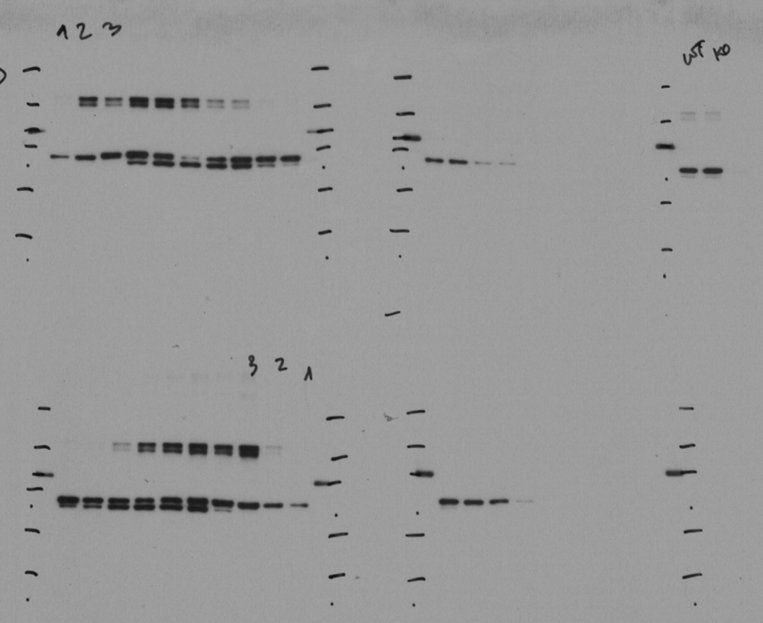

Supplement: Figure 3—source data 1. [file elife-83793-fig3-data1.zip › FIGURE3-source data/FIGURE3A/figure3Abip.jpg]

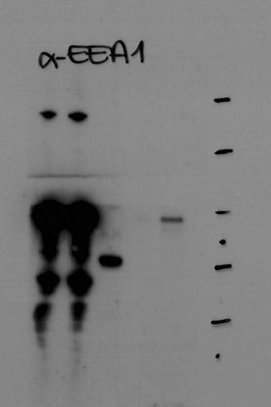

Supplement: Figure 3—source data 1. [file elife-83793-fig3-data1.zip › FIGURE3-source data/FIGURE3A/figure3BEEA1.jpg]

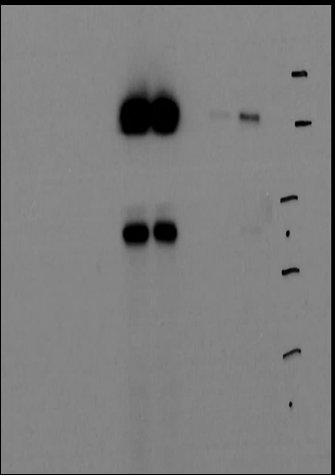

Supplement: Figure 3—source data 1. [file elife-83793-fig3-data1.zip › FIGURE3-source data/FIGURE3B/figure 3B gamma adaptin.jpg]

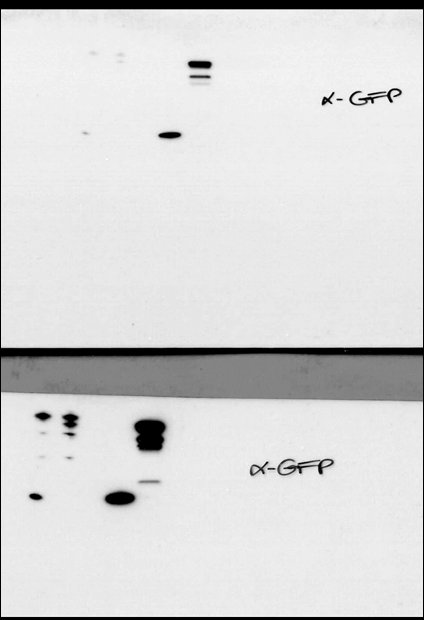

Supplement: Figure 3—source data 1. [file elife-83793-fig3-data1.zip › FIGURE3-source data/FIGURE3B/Figure 3B.GFP for clathrin EHD.jpg]

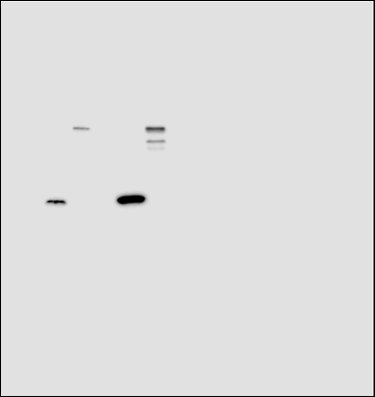

Supplement: Figure 3—source data 1. [file elife-83793-fig3-data1.zip › FIGURE3-source data/FIGURE3B/figure 3B.GFP for gamma adaptin.jpg]

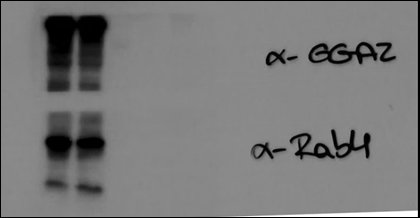

Supplement: Figure 3—source data 1. [file elife-83793-fig3-data1.zip › FIGURE3-source data/FIGURE3B/figure 3B.gga2.jpg]

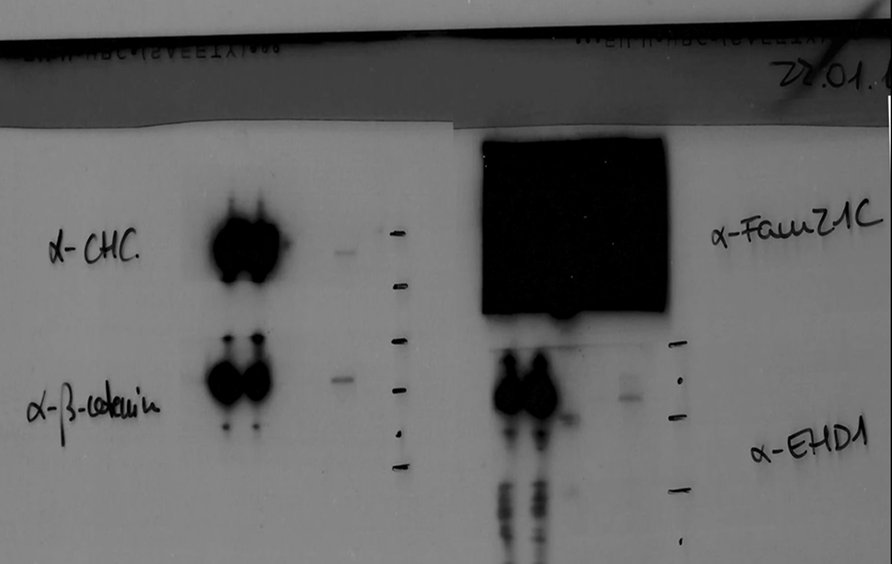

Supplement: Figure 3—source data 1. [file elife-83793-fig3-data1.zip › FIGURE3-source data/FIGURE3B/Figure3B.clathrin.jpg]

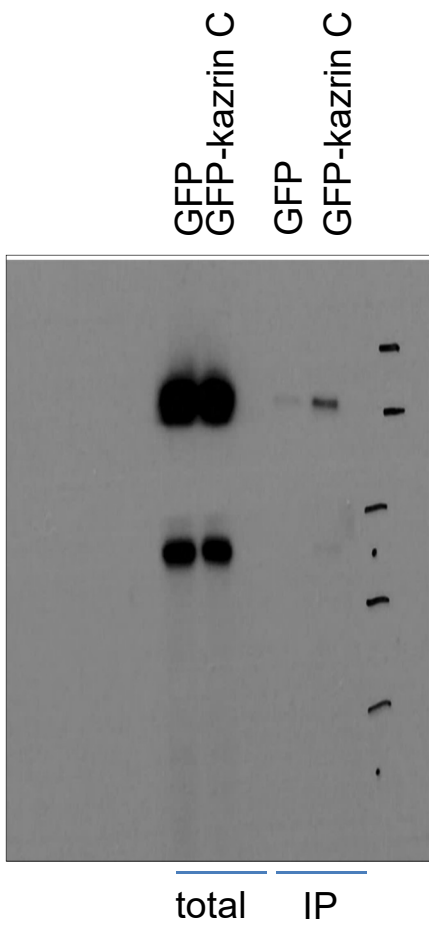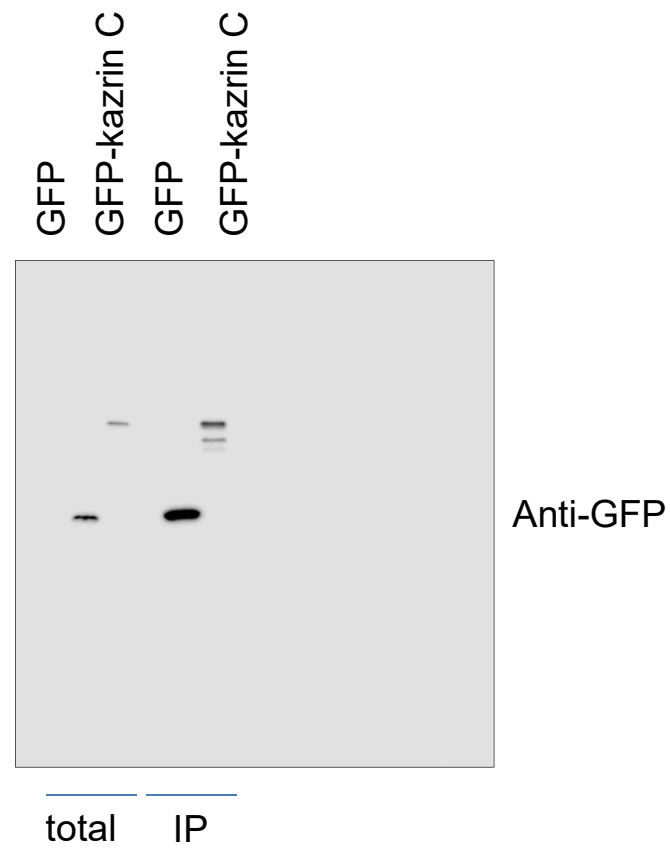

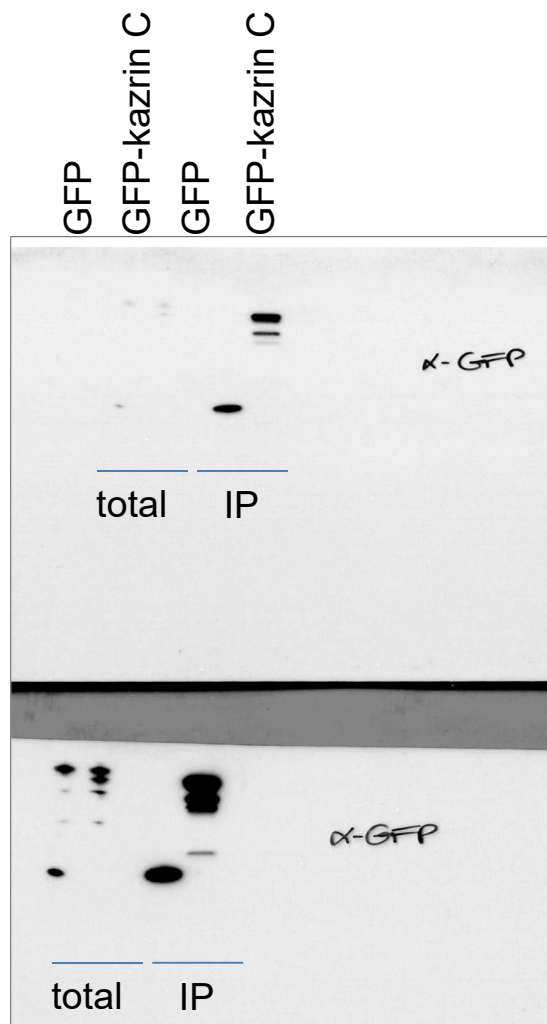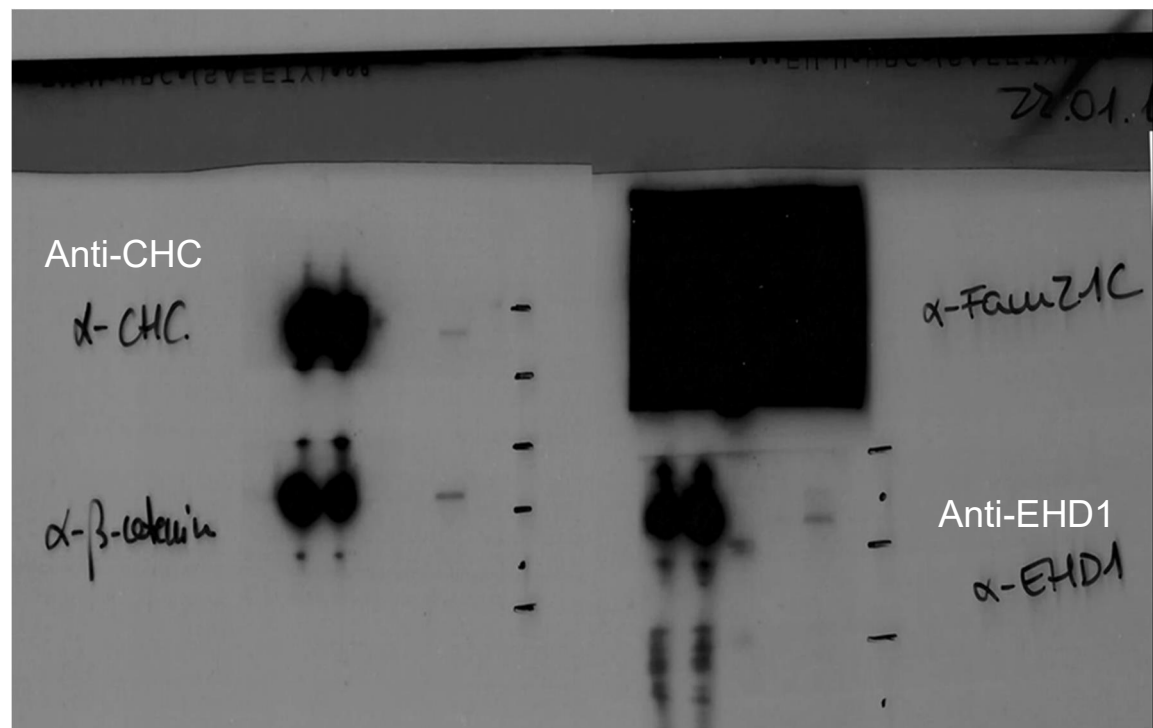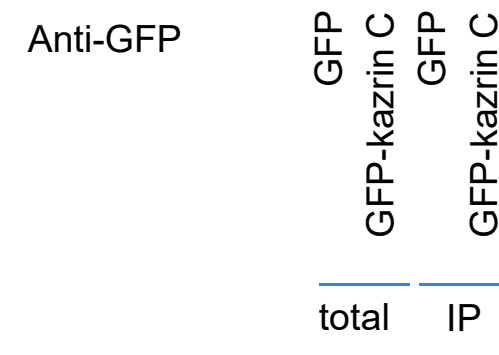

Anti-GFP

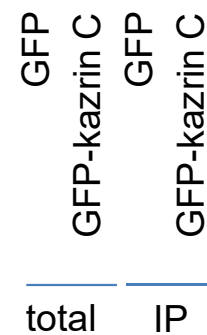

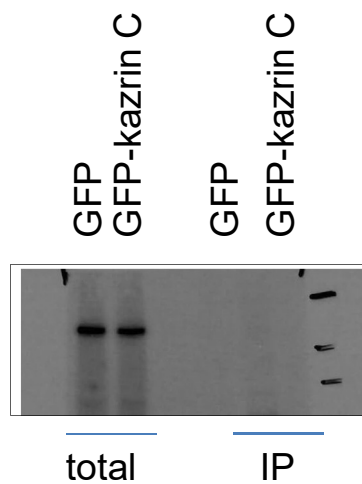

Anti-EEA1

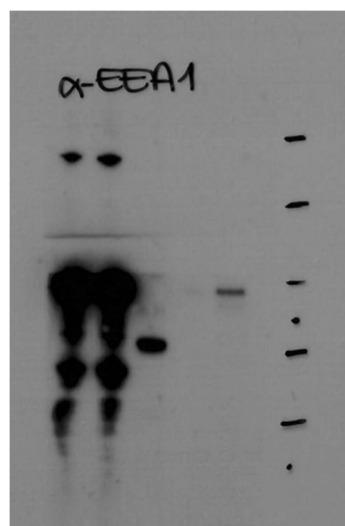

Anti-EEA1

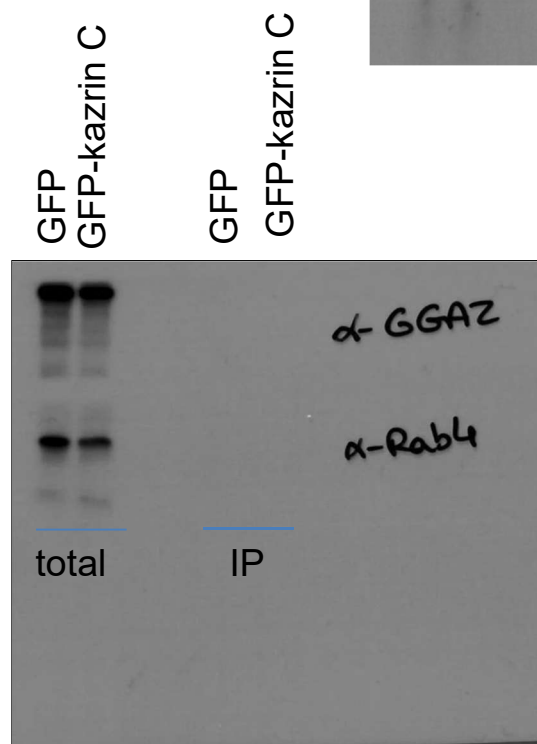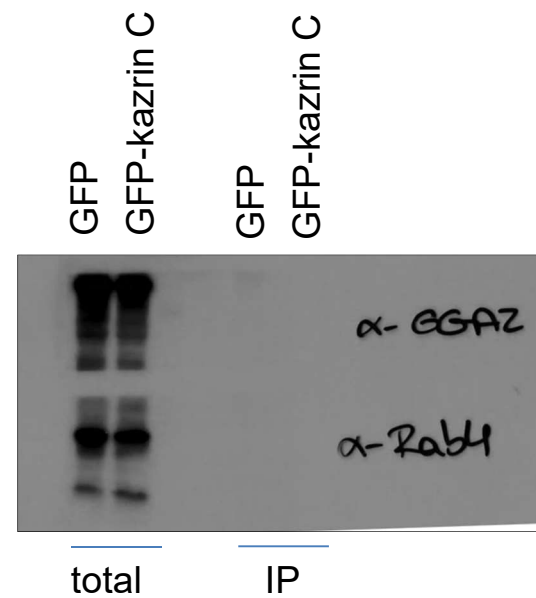

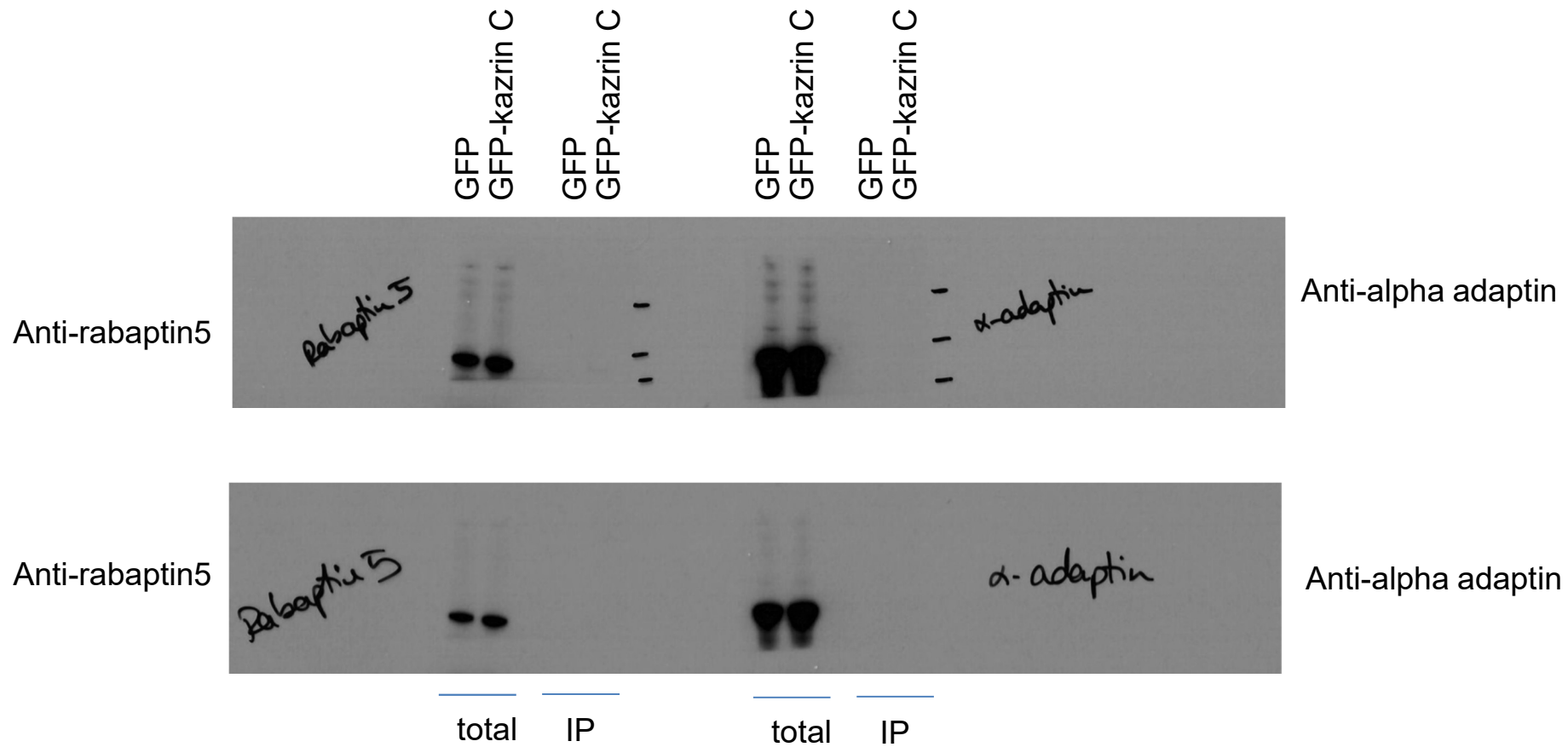

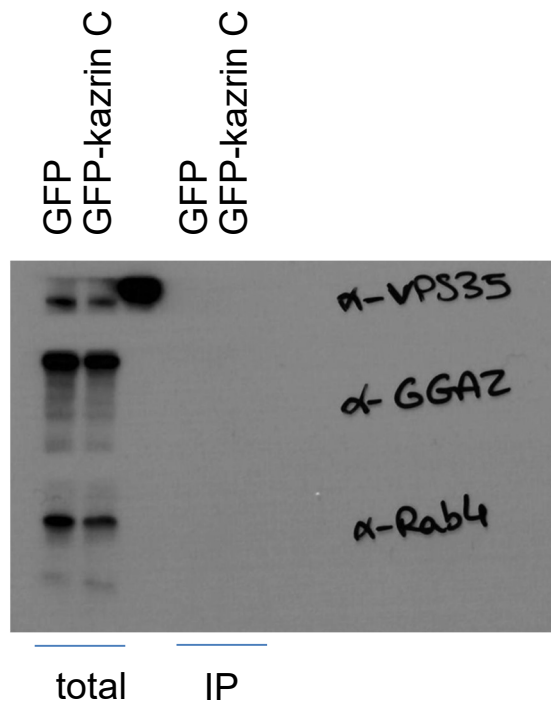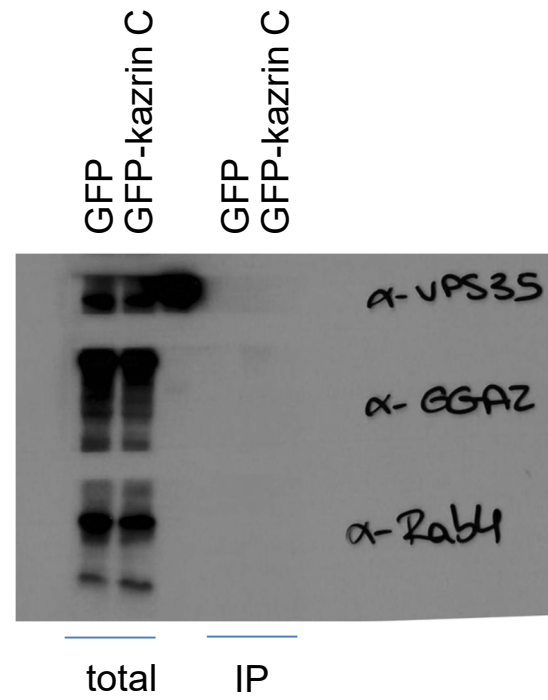

Supplement: Figure 3—source data 1. [file elife-83793-fig3-data1.zip › FIGURE3-source data/FIGURE3B/FIGURE3B.pdf]

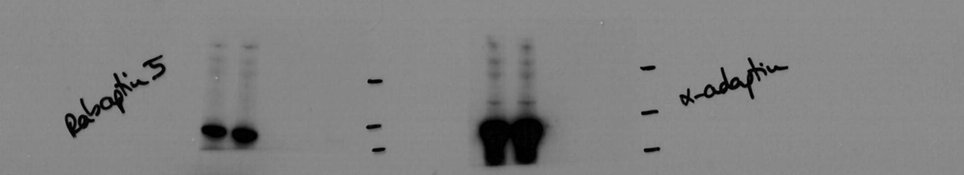

Supplement: Figure 3—source data 1. [file elife-83793-fig3-data1.zip › FIGURE3-source data/FIGURE3B/figure3B.rabaptin5.alpha-adaptin.jpg]

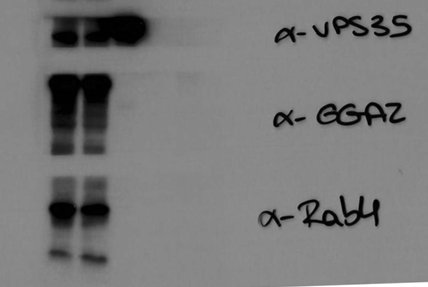

Supplement: Figure 3—source data 1. [file elife-83793-fig3-data1.zip › FIGURE3-source data/FIGURE3B/figure3B.vps35.jpg]

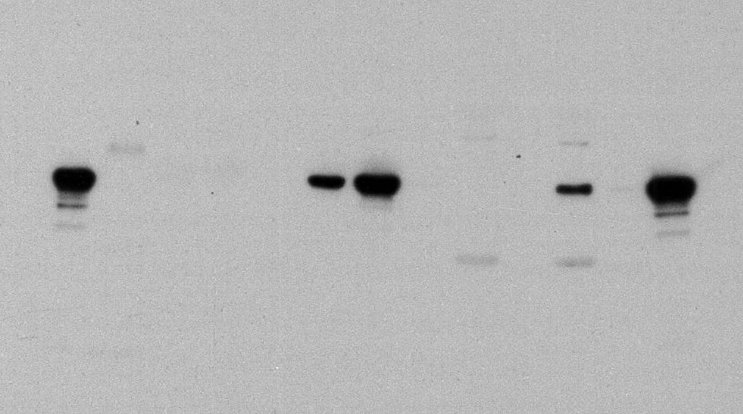

Supplement: Figure 3—source data 1. [file elife-83793-fig3-data1.zip › FIGURE3-source data/FIGURE3C/figure3C.clathrinTD.kazrin.jpg]

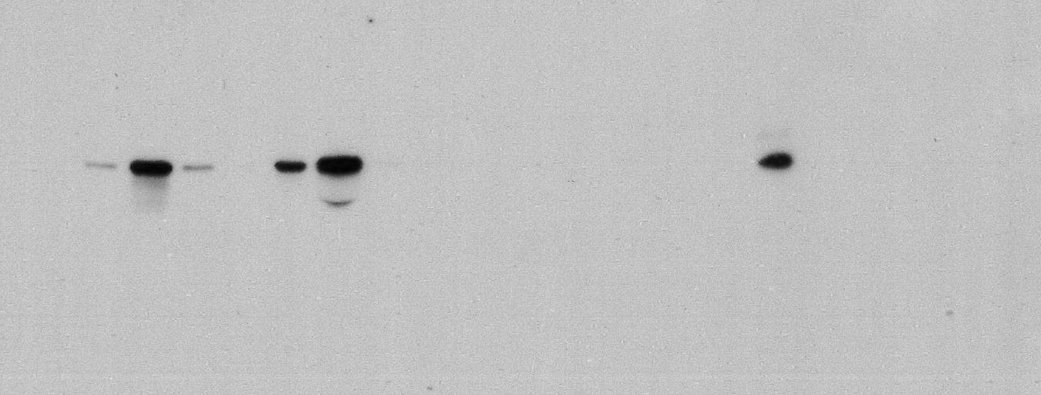

Supplement: Figure 3—source data 1. [file elife-83793-fig3-data1.zip › FIGURE3-source data/FIGURE3C/figure3C.gammaadaptin.kazrin.jpg]

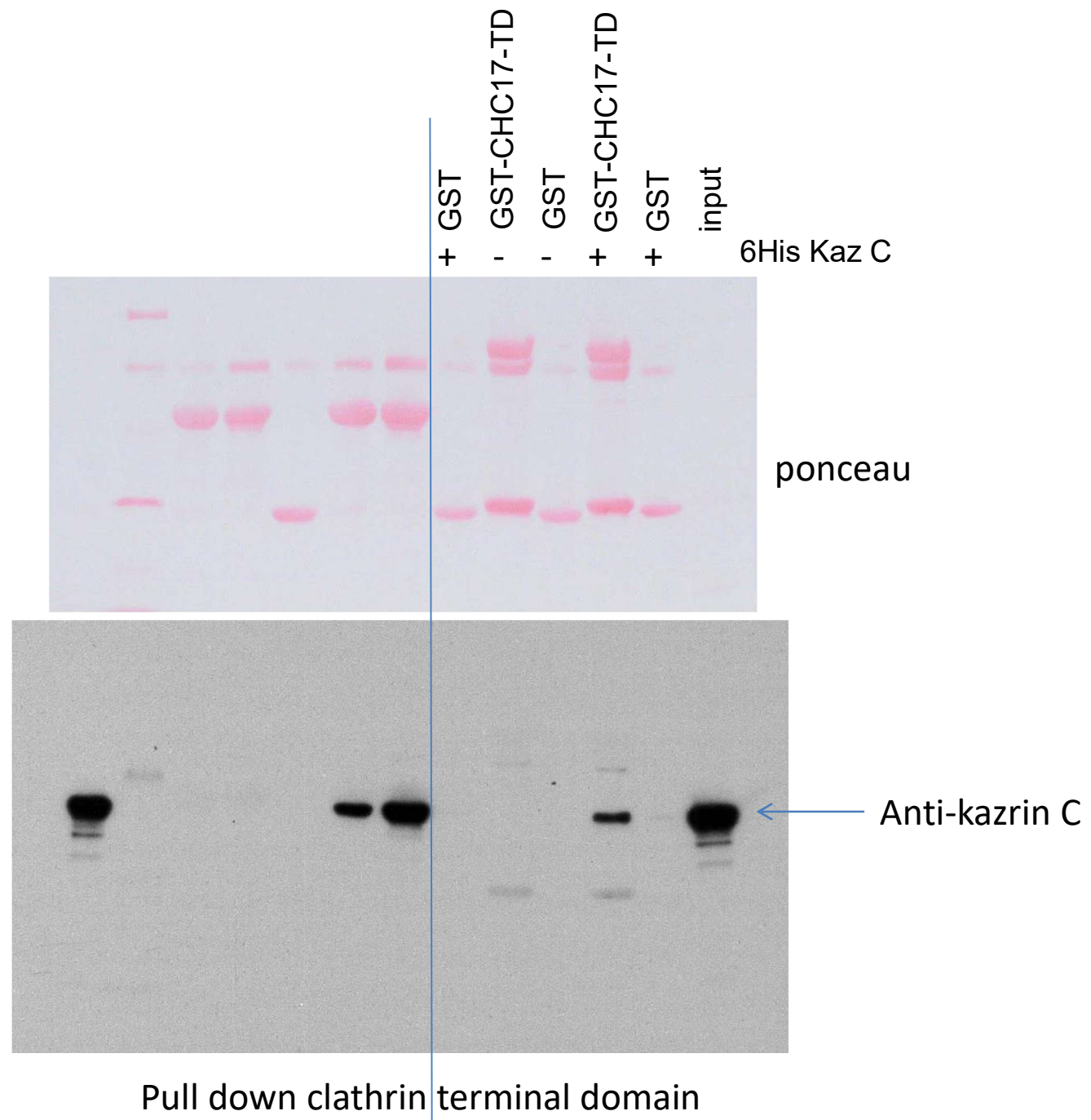

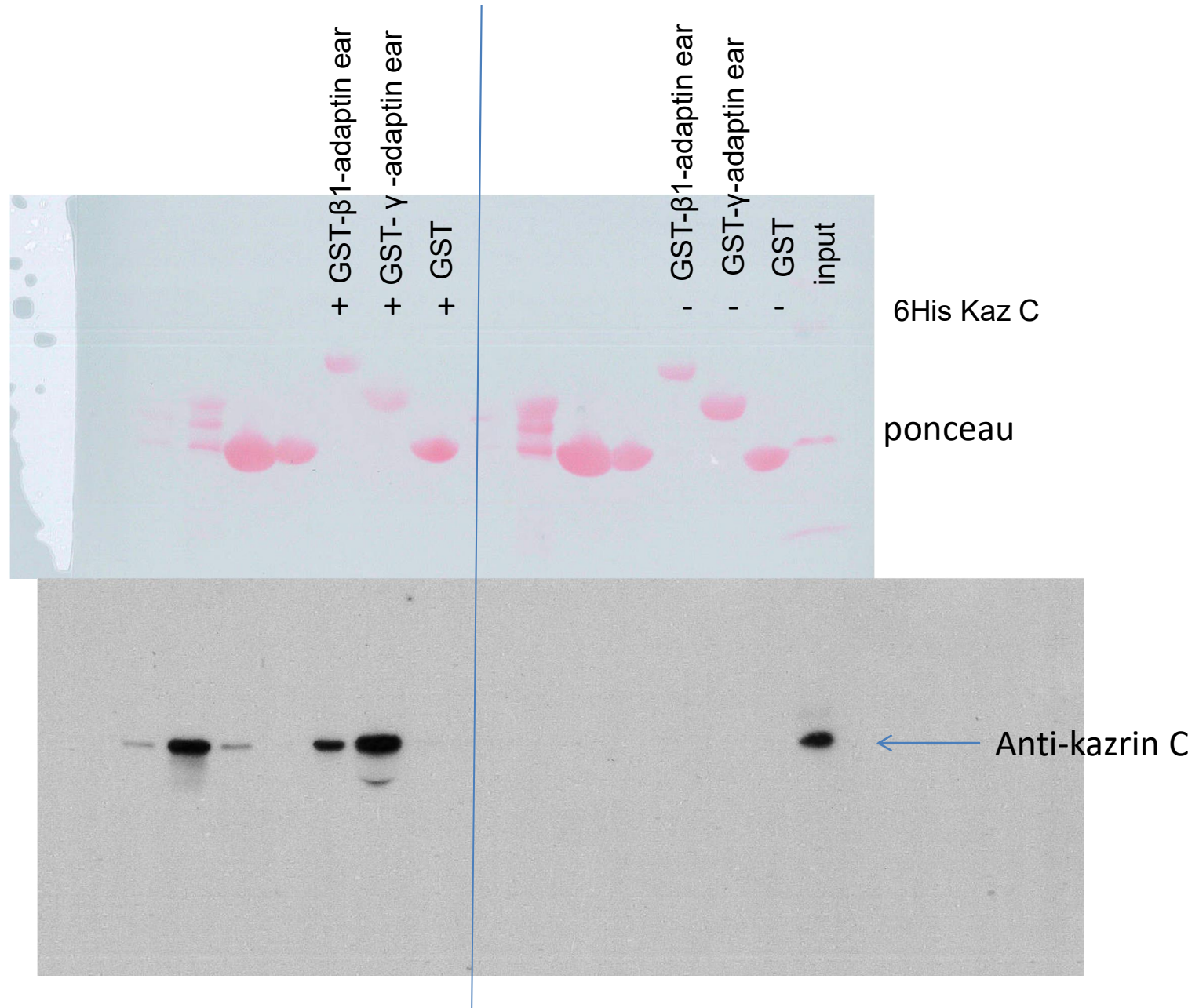

Pull down ear of gamma adaptin

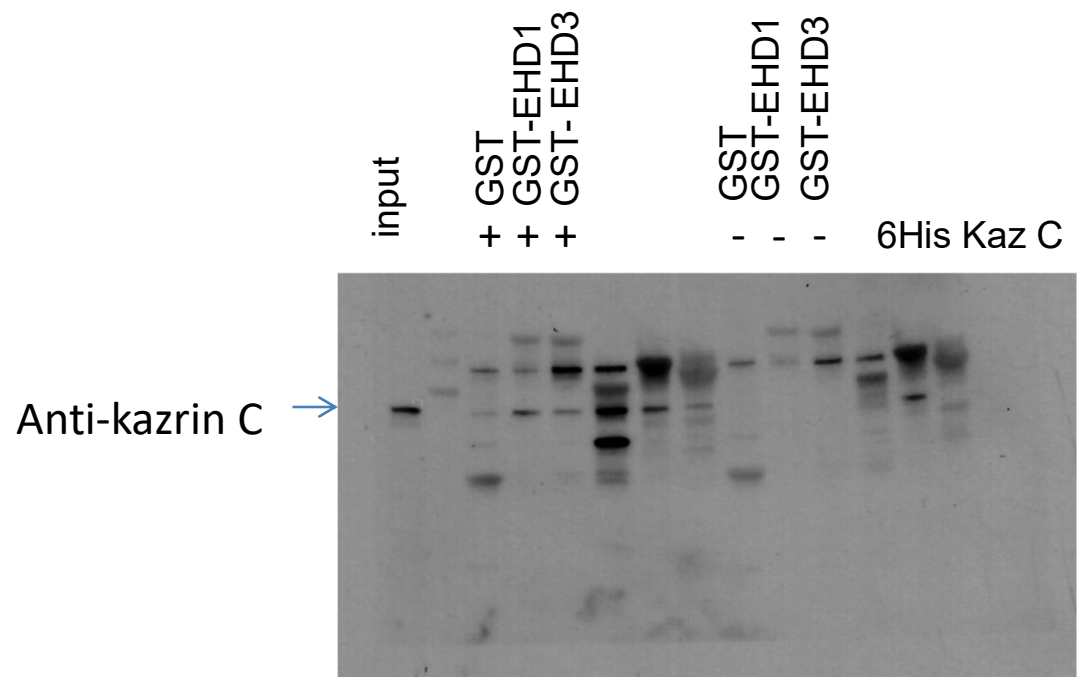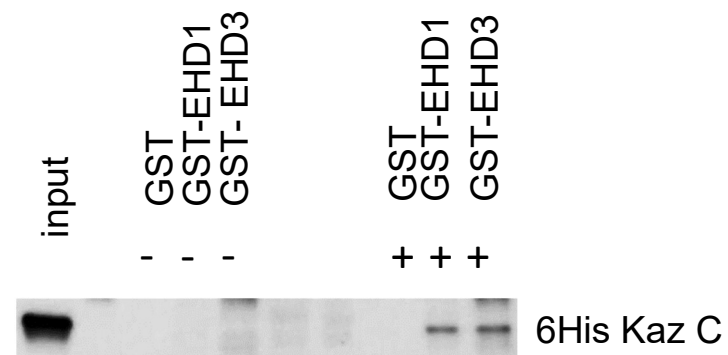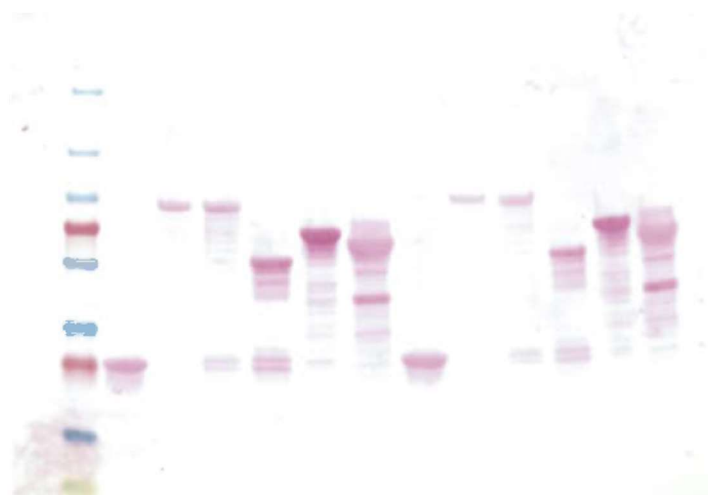

Primer exp

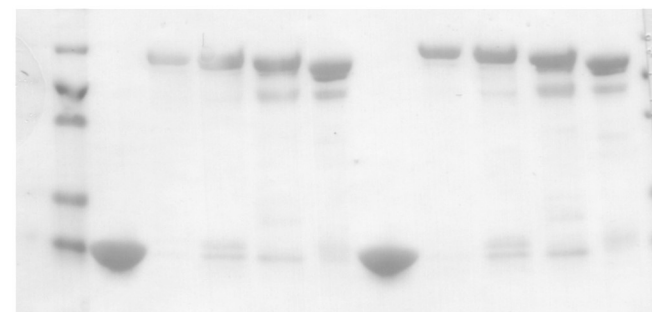

Segundo exp

Supplement: Figure 3—source data 1. [file elife-83793-fig3-data1.zip › FIGURE3-source data/FIGURE3C/FIGURE3C.pdf]

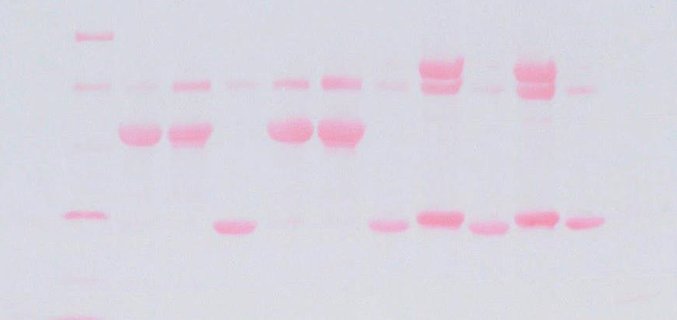

Supplement: Figure 3—source data 1. [file elife-83793-fig3-data1.zip › FIGURE3-source data/FIGURE3C/figure3Cponceau.clathrin terminal domain.jpg]

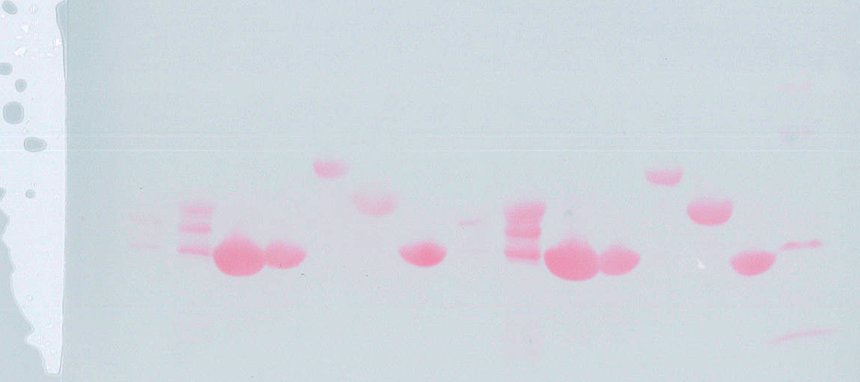

Supplement: Figure 3—source data 1. [file elife-83793-fig3-data1.zip › FIGURE3-source data/FIGURE3C/figure3Cponceau.gammaadaptin.jpg]

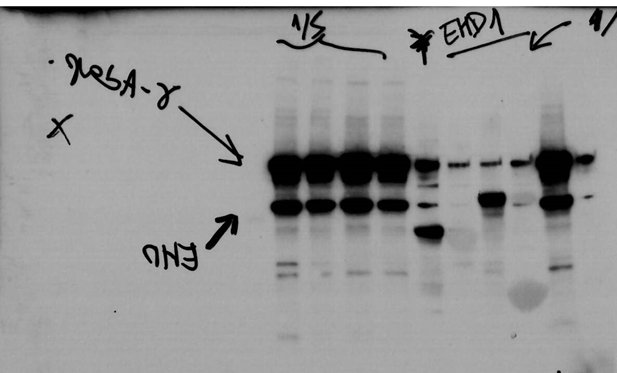

Supplement: Figure 3—source data 1. [file elife-83793-fig3-data1.zip › FIGURE3-source data/FIGURE3D/figure3DEHD1.jpg]

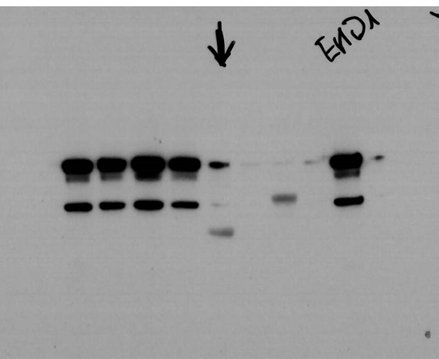

Supplement: Figure 3—source data 1. [file elife-83793-fig3-data1.zip › FIGURE3-source data/FIGURE3D/figure3Dgamma adaptin.jpg]

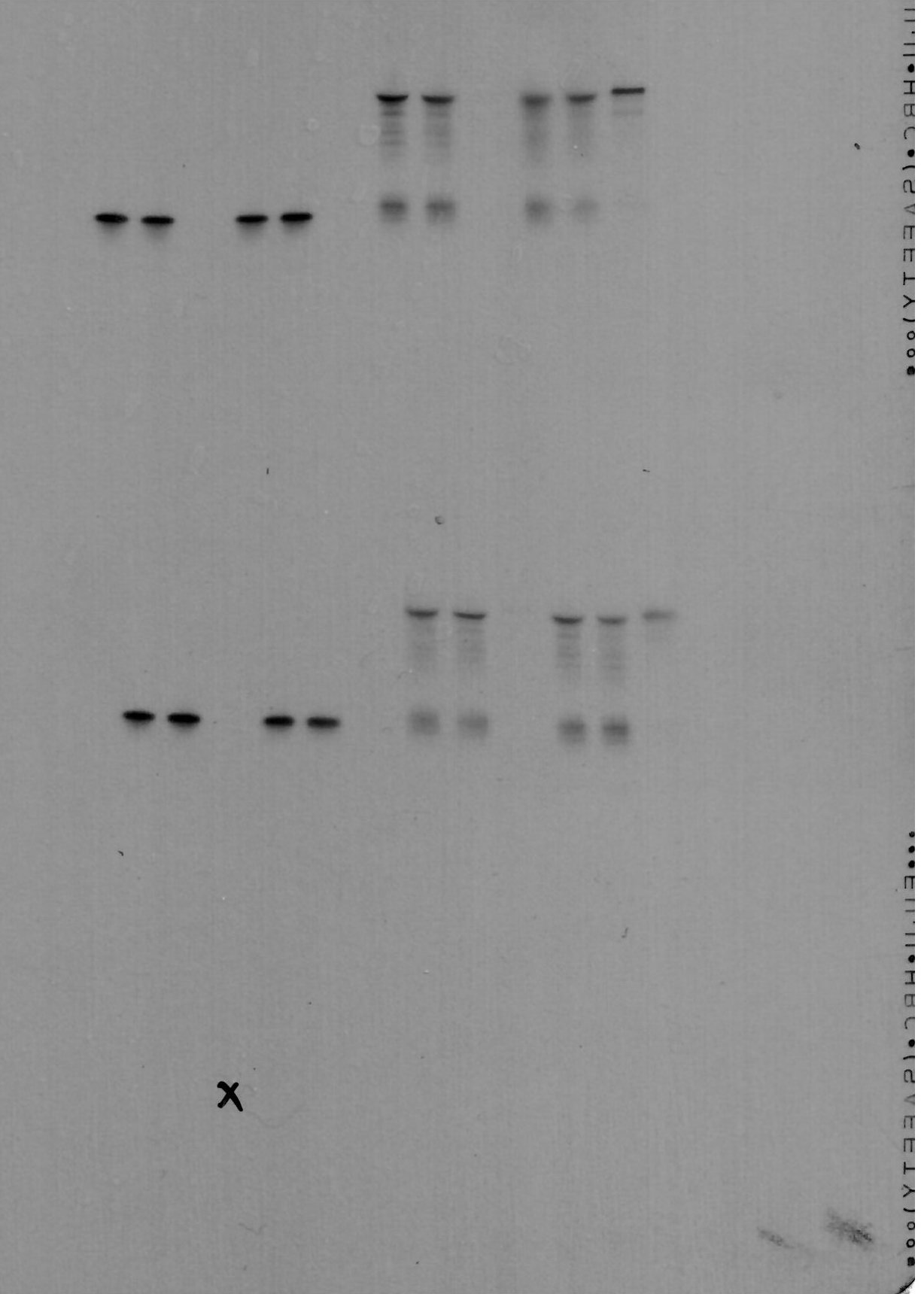

Supplement: Figure 3—source data 1. [file elife-83793-fig3-data1.zip › FIGURE3-source data/FIGURE3G/Figure 3G.antiGST.2.jpg]

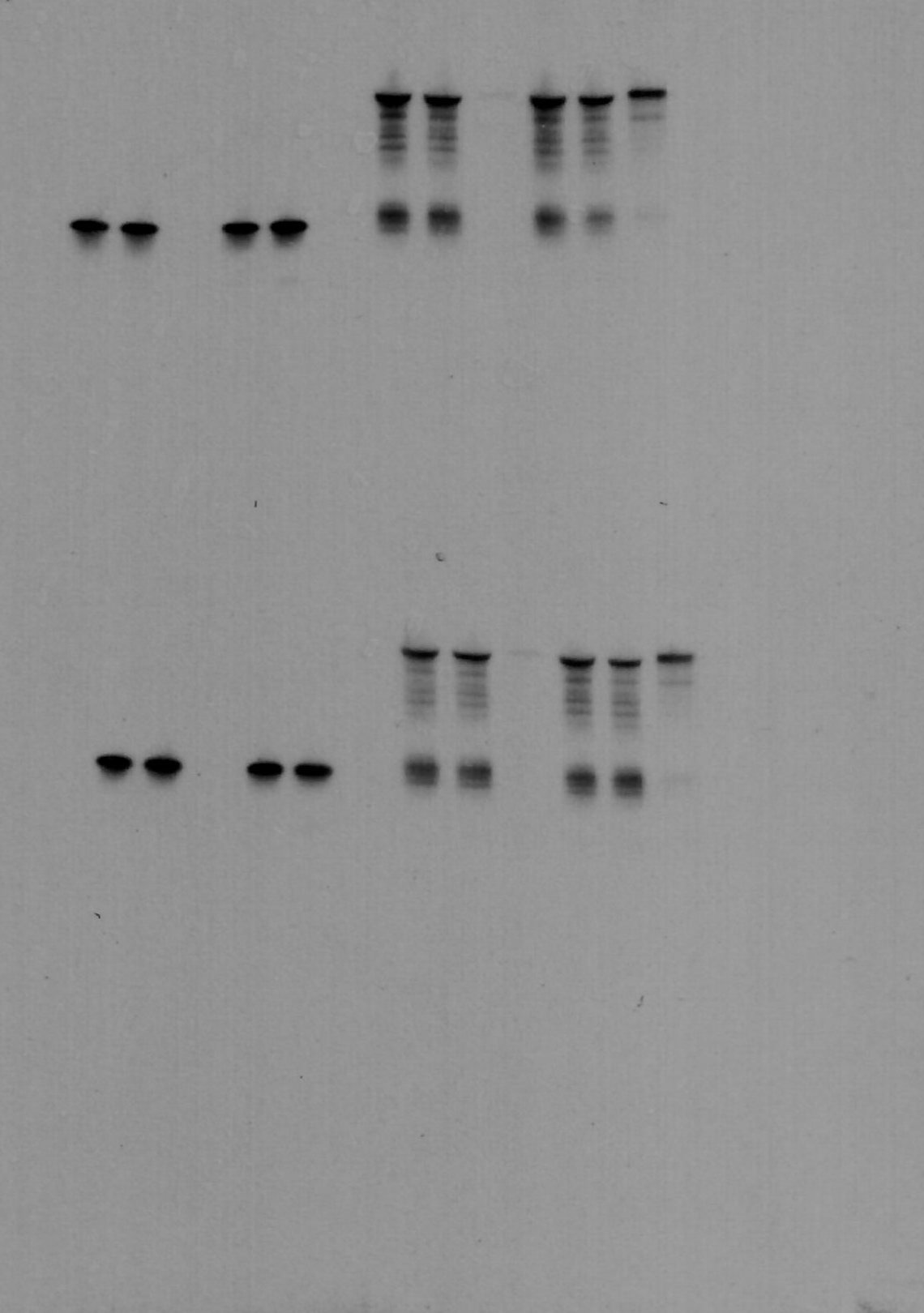

Supplement: Figure 3—source data 1. [file elife-83793-fig3-data1.zip › FIGURE3-source data/FIGURE3G/figure3G.anti GST.jpg]

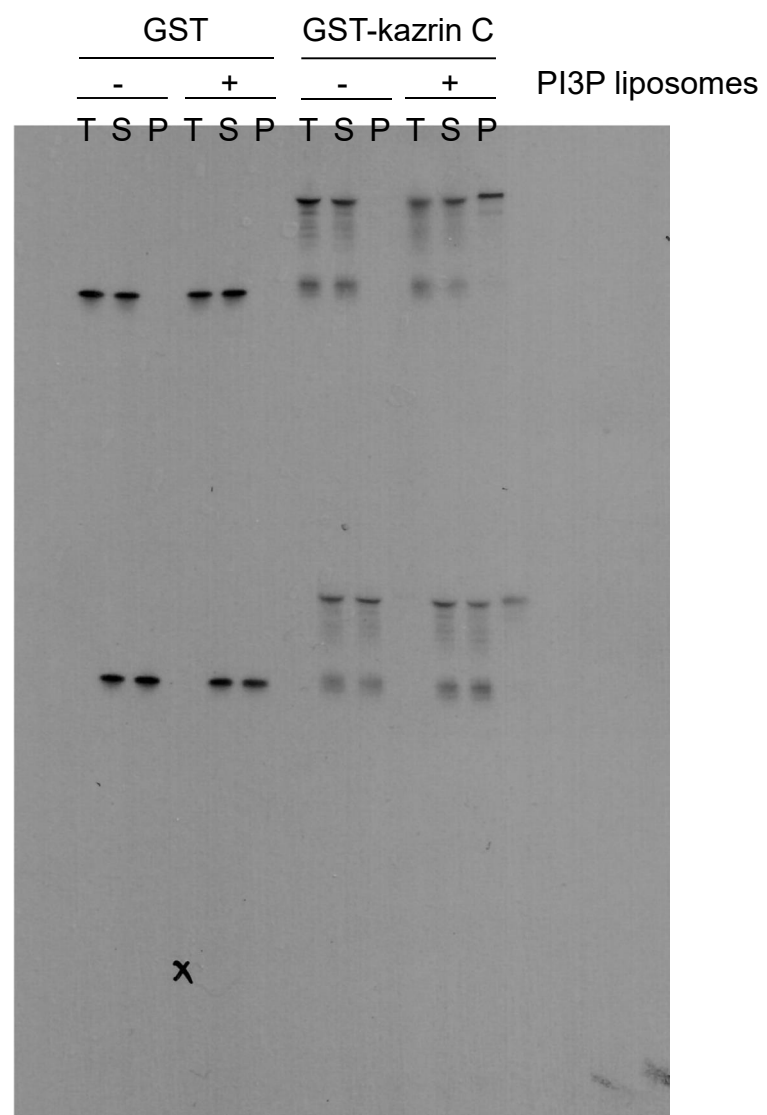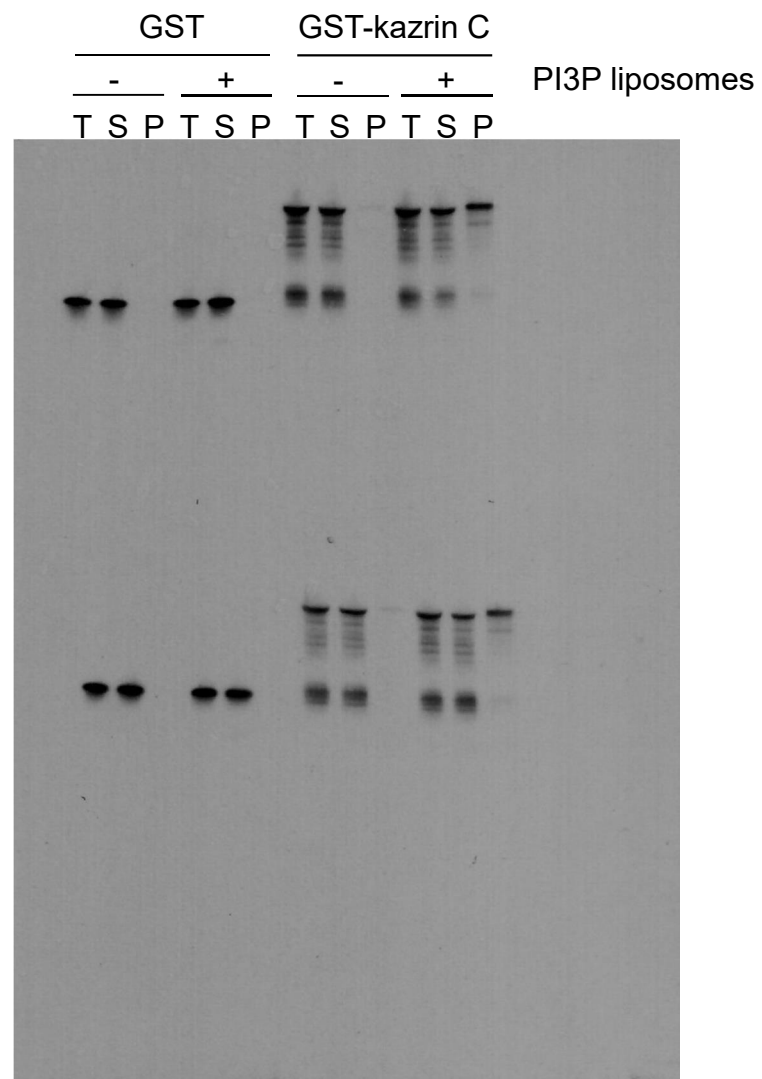

Supplement: Figure 3—source data 1. [file elife-83793-fig3-data1.zip › FIGURE3-source data/FIGURE3G/FIGURE3G.pdf]

# Opiprep density gradient

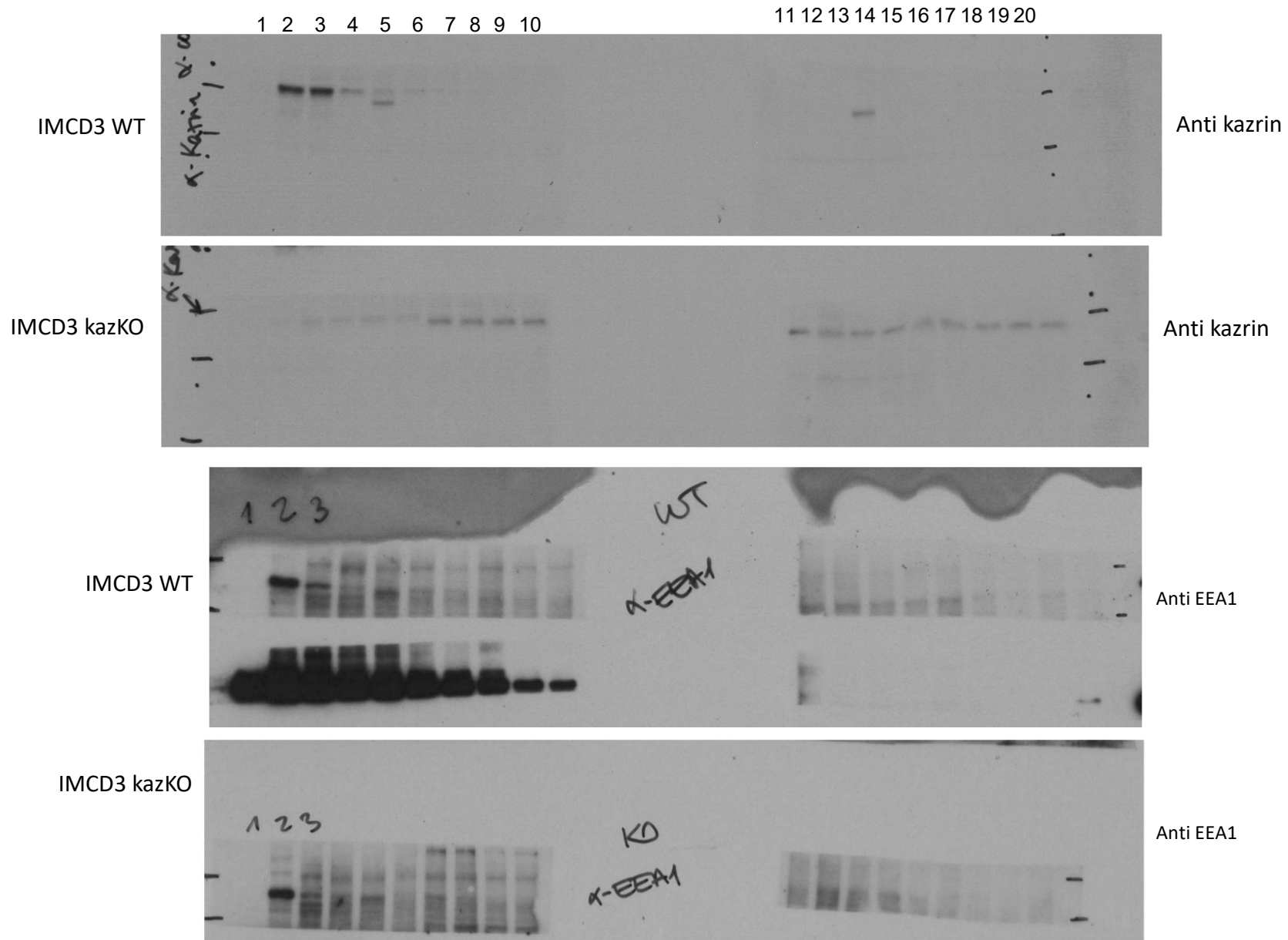

Supplement: Figure 3—figure supplement 1—source data 1. [file elife-83793-fig3-figsupp1-data1.zip › FIGURE3-figure supplement1-source data/FIGURE3-figure supplement1.pdf]

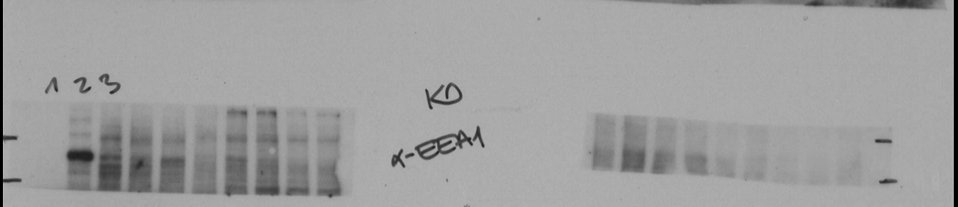

Supplement: Figure 3—figure supplement 1—source data 1. [file elife-83793-fig3-figsupp1-data1.zip › FIGURE3-figure supplement1-source data/figure3-S1.KO.EEA1.jpg]

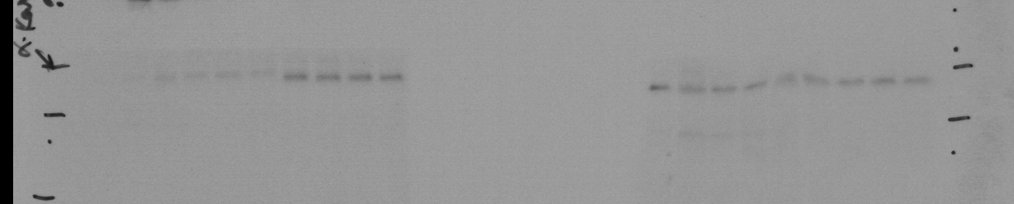

Supplement: Figure 3—figure supplement 1—source data 1. [file elife-83793-fig3-figsupp1-data1.zip › FIGURE3-figure supplement1-source data/figure3-S1.KO.kazrin.jpg]

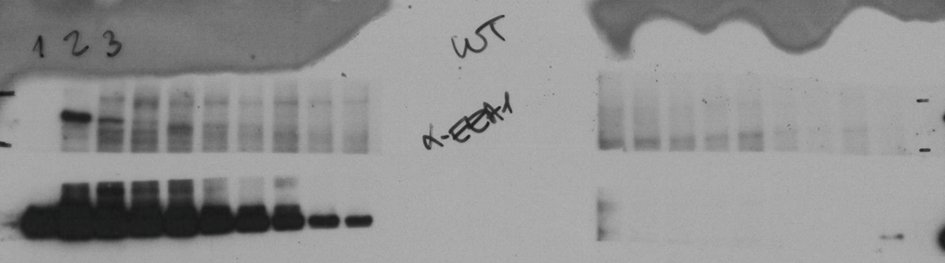

Supplement: Figure 3—figure supplement 1—source data 1. [file elife-83793-fig3-figsupp1-data1.zip › FIGURE3-figure supplement1-source data/figure3-S1.WT.EEA1.jpg]

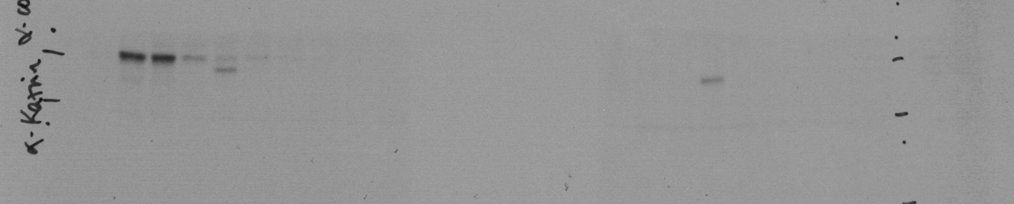

Supplement: Figure 3—figure supplement 1—source data 1. [file elife-83793-fig3-figsupp1-data1.zip › FIGURE3-figure supplement1-source data/figure3-S1.WT.kazrin.jpg]

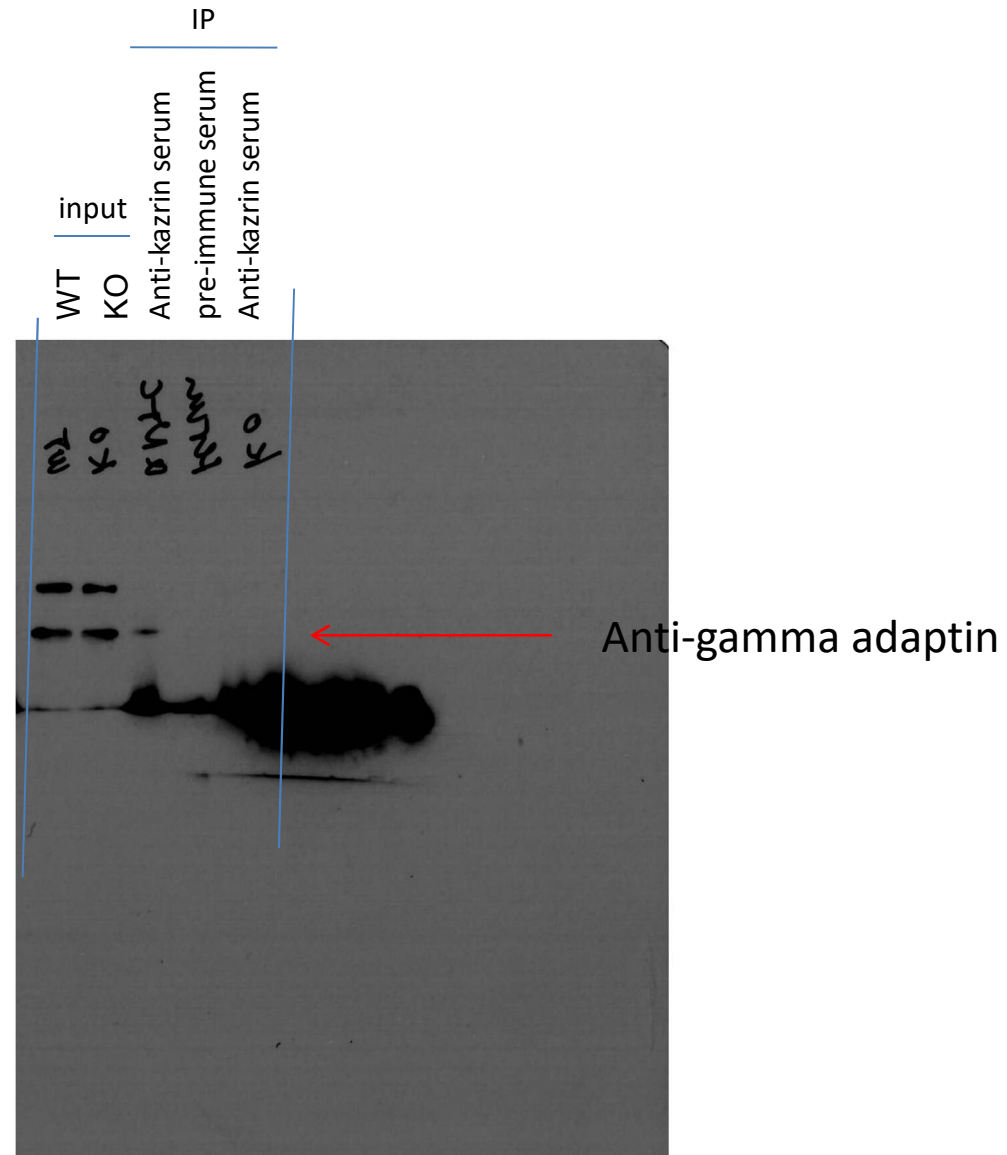

Supplement: Figure 3—figure supplement 2—source data 1. [file elife-83793-fig3-figsupp2-data1.zip › FIGURE3-figure supplement2-source data/FIGURE3-figure supplement2.pdf]

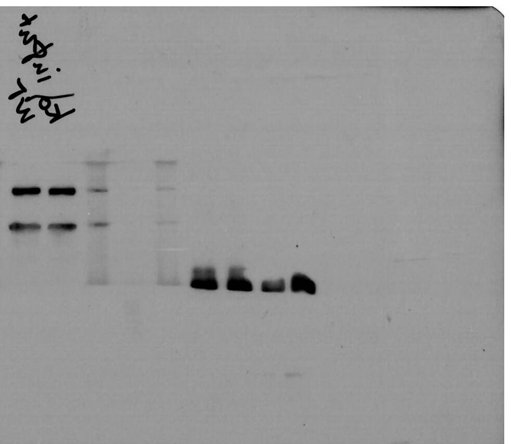

Supplement: Figure 3—figure supplement 2—source data 1. [file elife-83793-fig3-figsupp2-data1.zip › FIGURE3-figure supplement2-source data/figure3-S2clathrin.jpg]

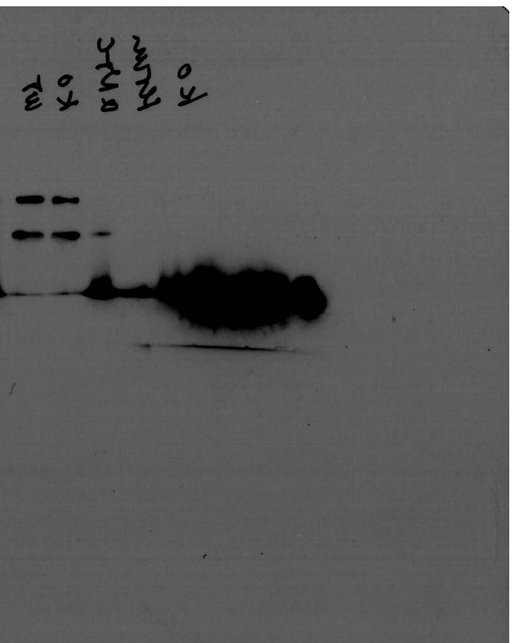

Supplement: Figure 3—figure supplement 2—source data 1. [file elife-83793-fig3-figsupp2-data1.zip › FIGURE3-figure supplement2-source data/figure3-S2gammaadaptin.jpg]

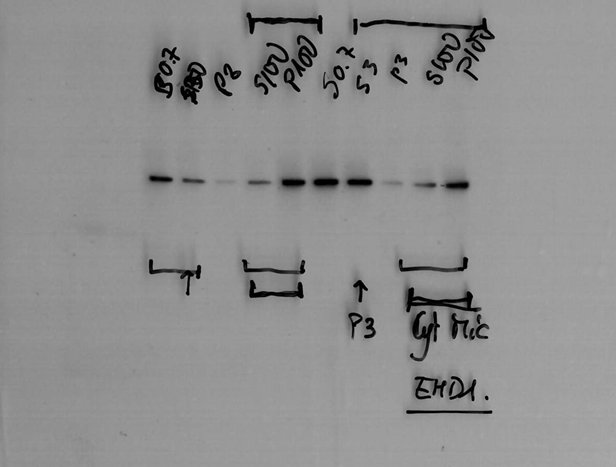

Supplement: Figure 4—source data 1. [file elife-83793-fig4-data1.zip › FIGURE4-source data1/figure4A.GFPkaz.GFP.antiEHD.jpg]

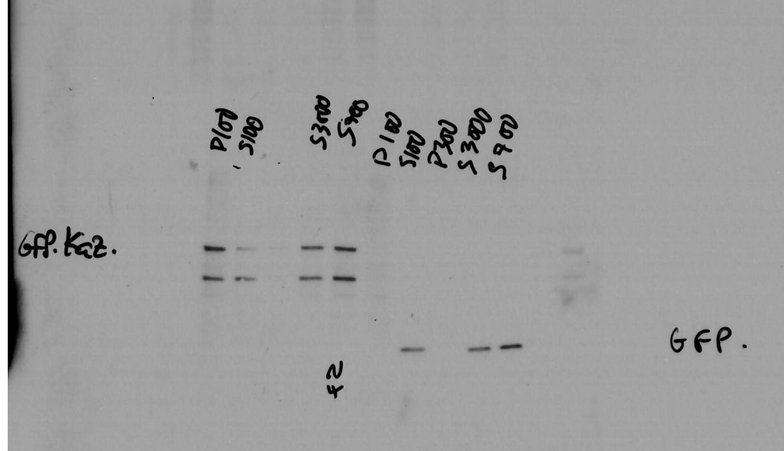

Supplement: Figure 4—source data 1. [file elife-83793-fig4-data1.zip › FIGURE4-source data1/figure4A.GFPkaz.GFP.antiGFP.jpg]

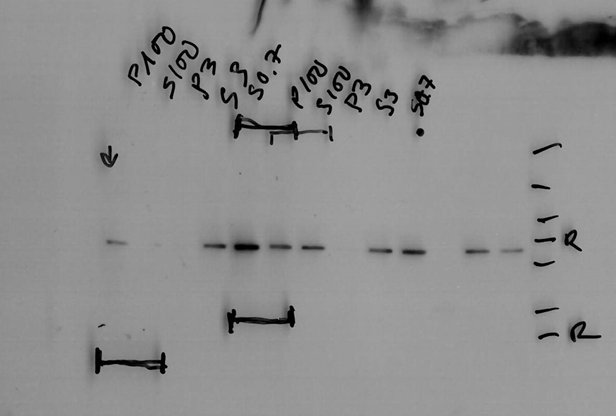

Supplement: Figure 4—source data 1. [file elife-83793-fig4-data1.zip › FIGURE4-source data1/figure4A.GFPkazNt.antiEHD1.jpg]

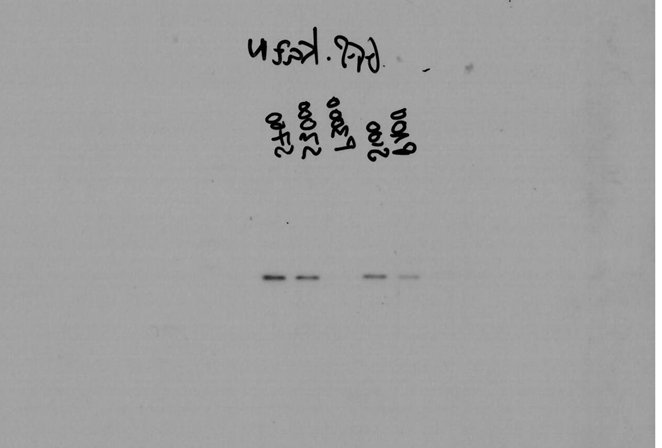

Supplement: Figure 4—source data 1. [file elife-83793-fig4-data1.zip › FIGURE4-source data1/figure4A.GFPkazNt.antiGFP.jpg]

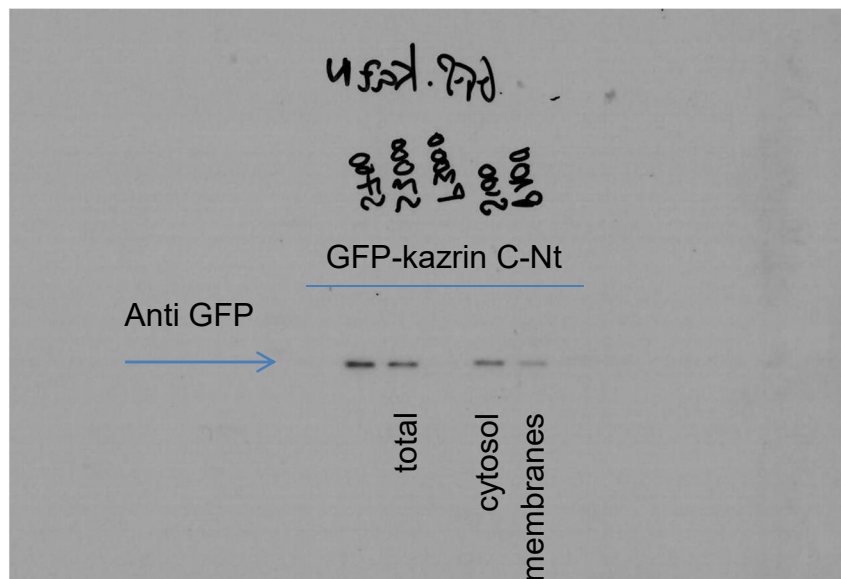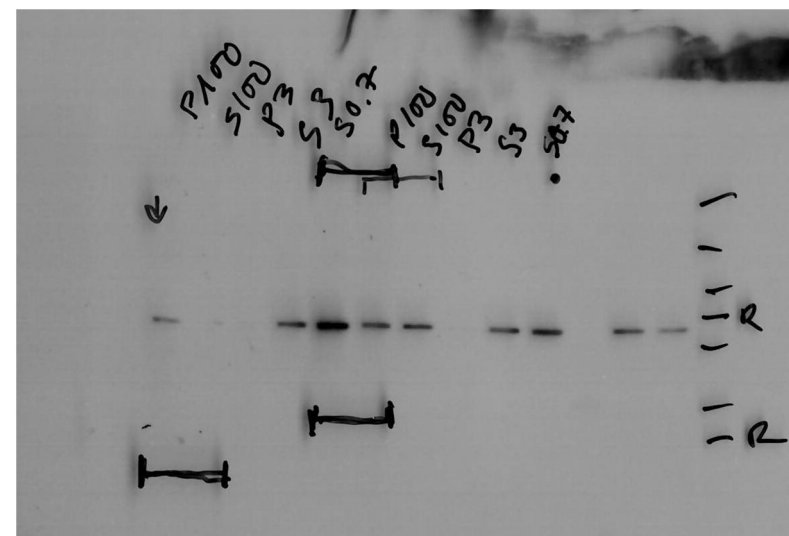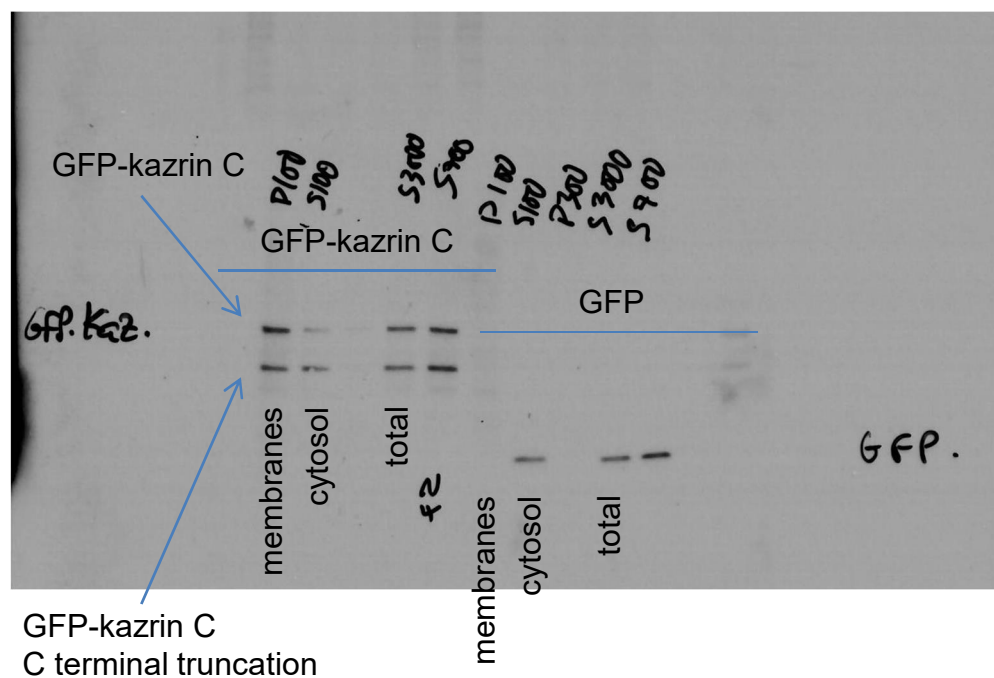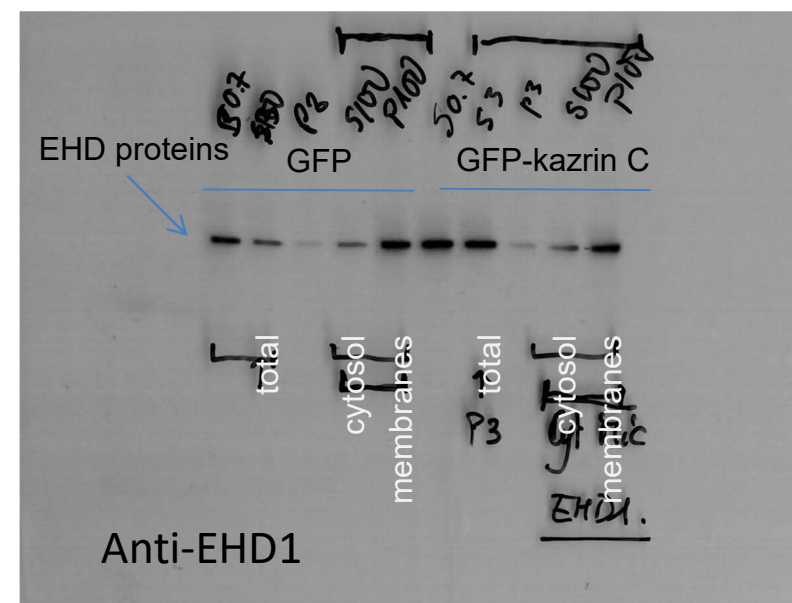

Supplement: Figure 4—source data 1. [file elife-83793-fig4-data1.zip › FIGURE4-source data1/FIGURE4A.pdf]

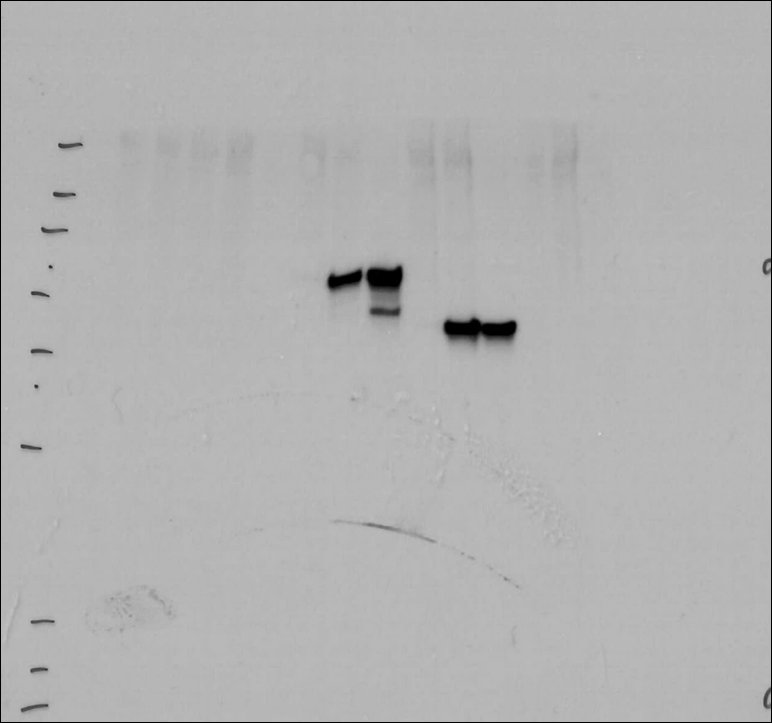

Supplement: Figure 4—figure supplement 1—source data 1. [file elife-83793-fig4-figsupp1-data1.zip › FIGURE4-figure supplement1-source data/figure4-S1.GFP.jpg]

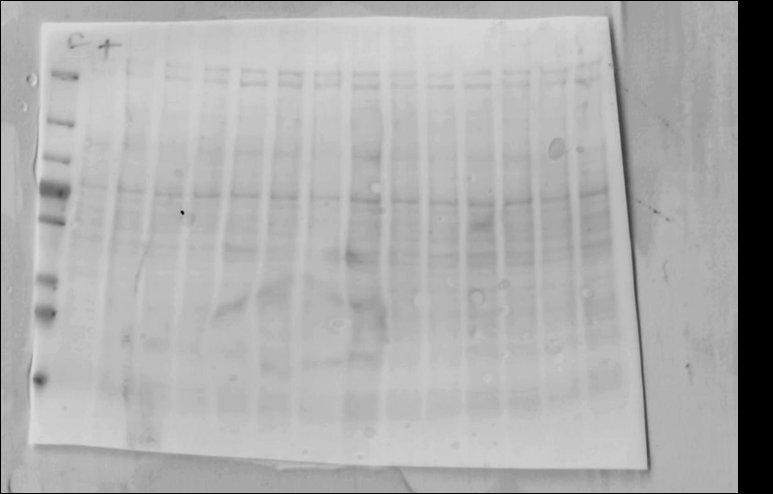

Supplement: Figure 4—figure supplement 1—source data 1. [file elife-83793-fig4-figsupp1-data1.zip › FIGURE4-figure supplement1-source data/figure4-S1.ponceau.jpg]

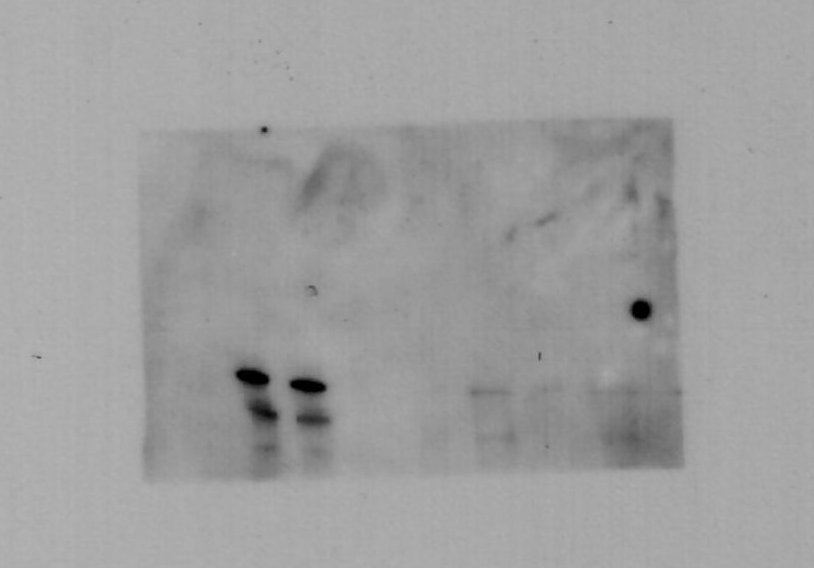

Supplement: Figure 5—source data 1. [file elife-83793-fig5-data1.zip › FIGURE5-source data1/FIGURE5H/Dynein IP,anti dynein HC.2.jpg]

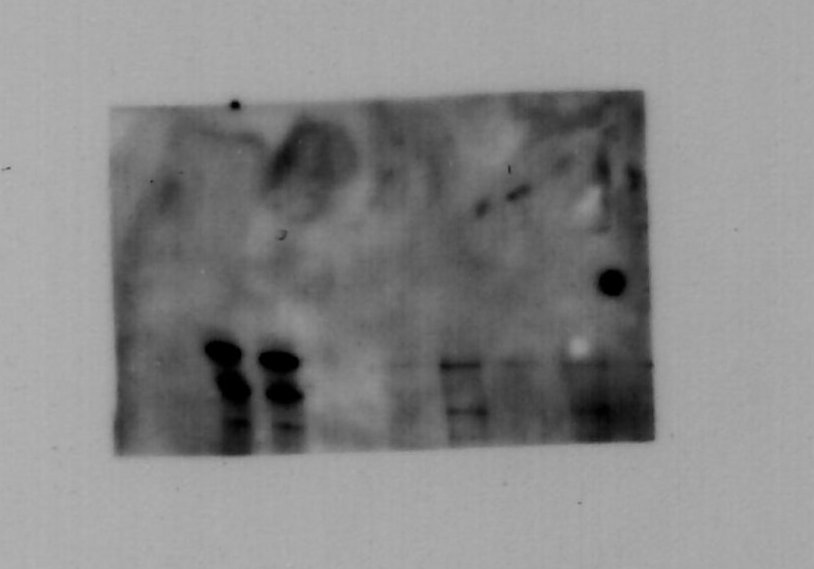

Supplement: Figure 5—source data 1. [file elife-83793-fig5-data1.zip › FIGURE5-source data1/FIGURE5H/Dynein IP,anti dynein HC.jpg]

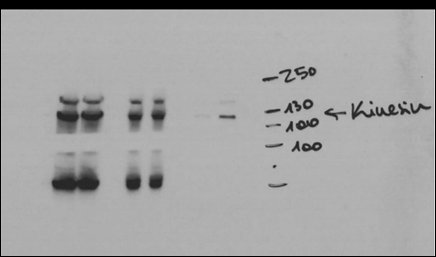

Supplement: Figure 5—source data 1. [file elife-83793-fig5-data1.zip › FIGURE5-source data1/FIGURE5H/figure5F.antitkinesin1.jpg]

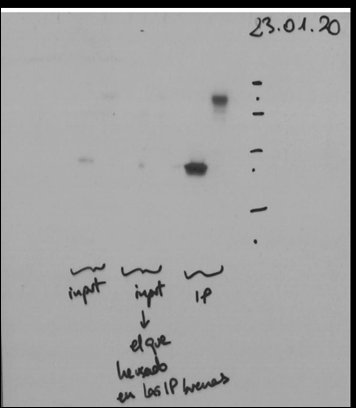

Supplement: Figure 5—source data 1. [file elife-83793-fig5-data1.zip › FIGURE5-source data1/FIGURE5H/figure5H.antitGFP.jpg]

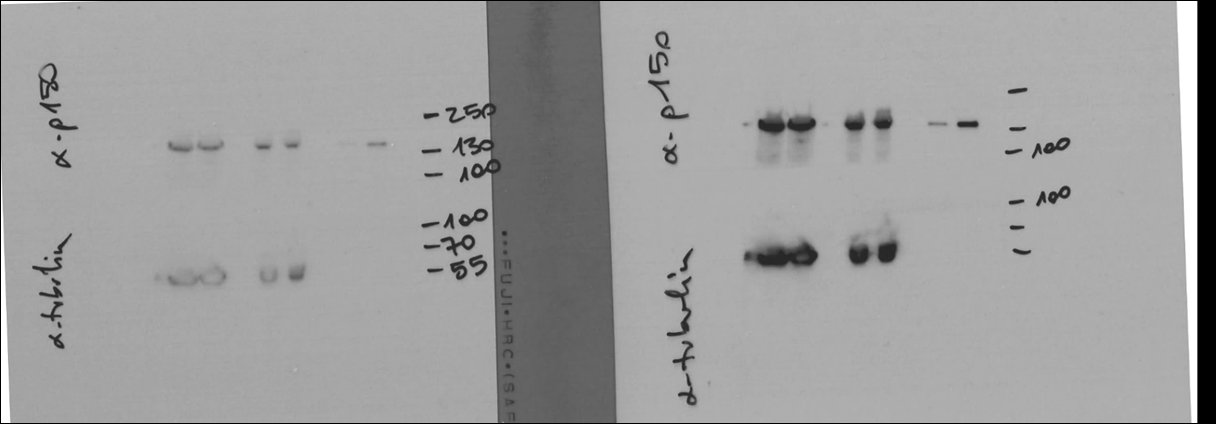

Supplement: Figure 5—source data 1. [file elife-83793-fig5-data1.zip › FIGURE5-source data1/FIGURE5H/figure5H.antitubulin.antip150glued.jpg]

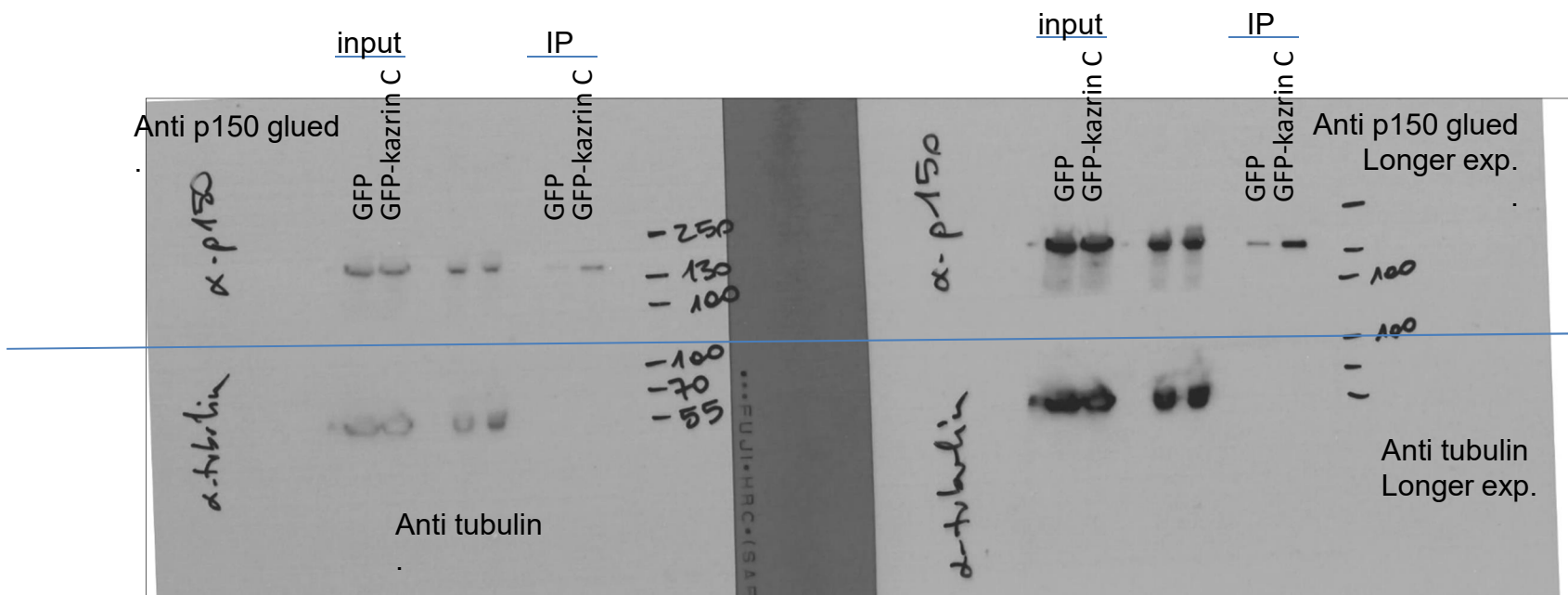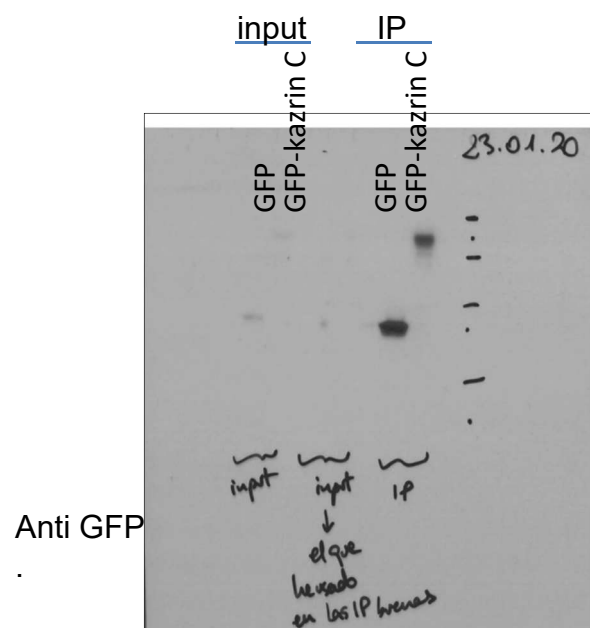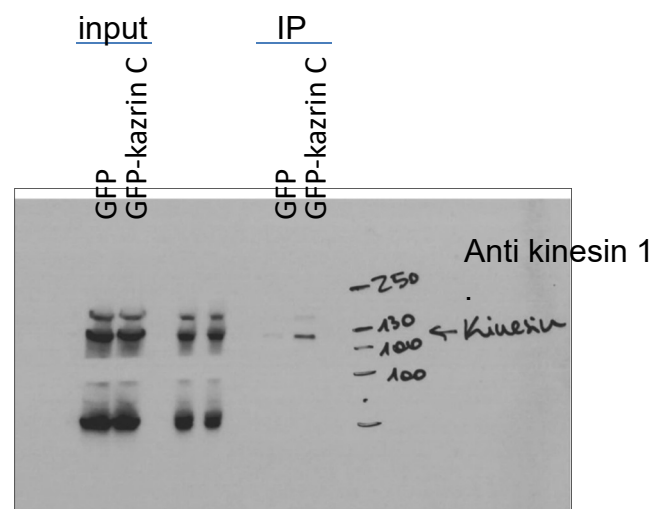

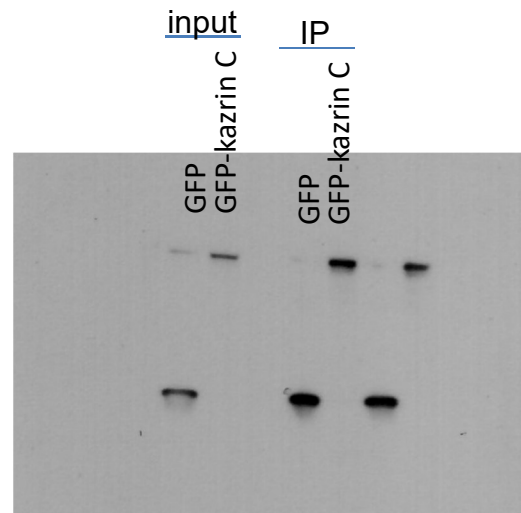

Anti GFP

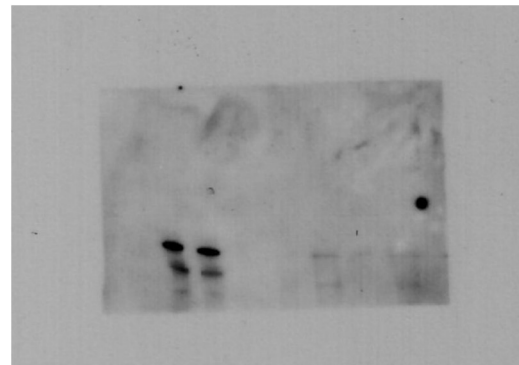

Anti Dynein HC

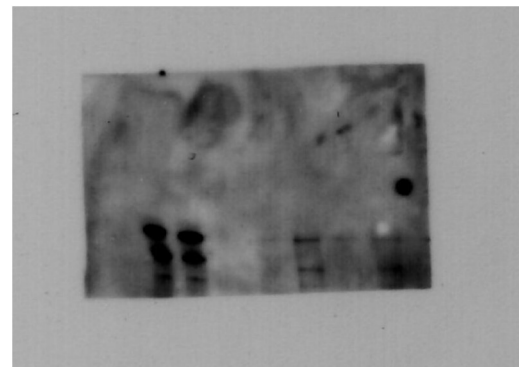

Anti Dynein HC longer exposure

Supplement: Figure 5—source data 1. [file elife-83793-fig5-data1.zip › FIGURE5-source data1/FIGURE5H/FIGURE5H.pdf]

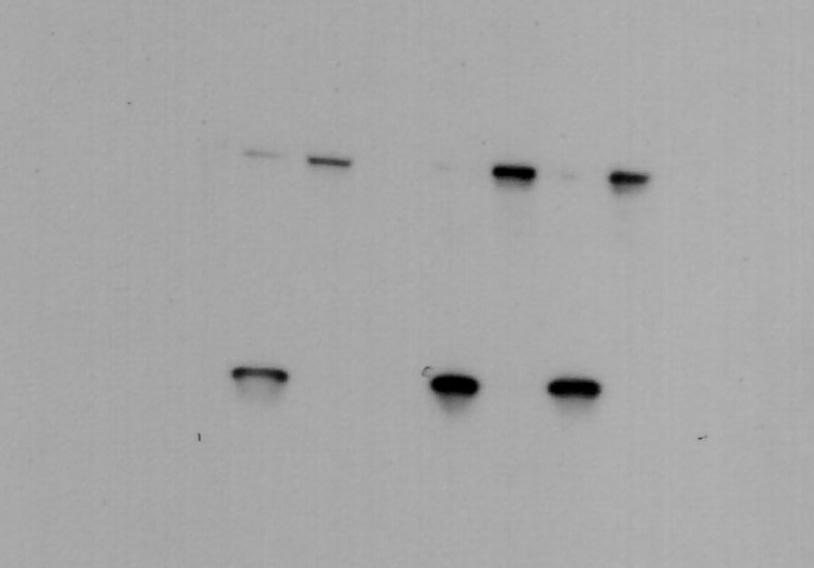

Supplement: Figure 5—source data 1. [file elife-83793-fig5-data1.zip › FIGURE5-source data1/FIGURE5H/IP dynein. anti GFP.tif]

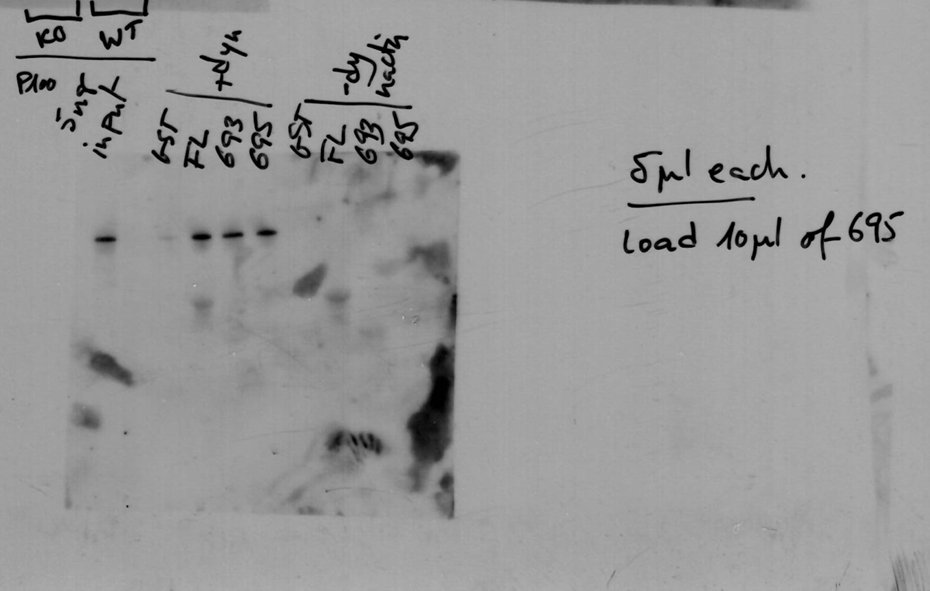

Supplement: Figure 5—source data 1. [file elife-83793-fig5-data1.zip › FIGURE5-source data1/FIGURE5I/figure5I.antip150.jpg]

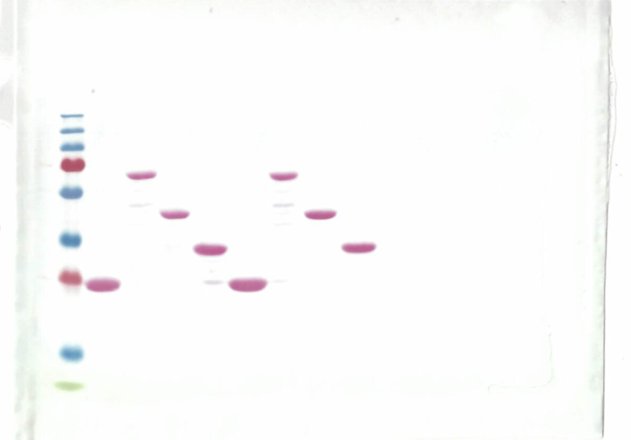

Supplement: Figure 5—source data 1. [file elife-83793-fig5-data1.zip › FIGURE5-source data1/FIGURE5I/figure5I.ponceau.jpg]

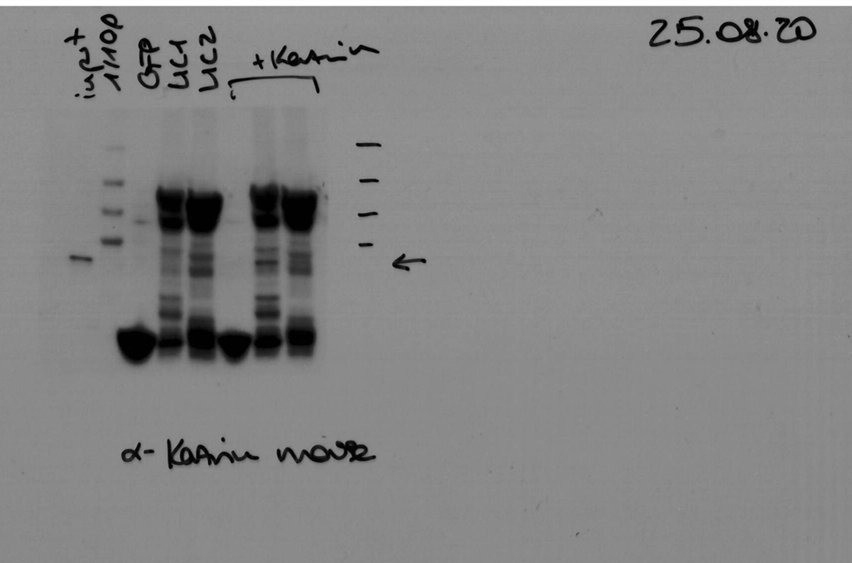

Supplement: Figure 5—source data 1. [file elife-83793-fig5-data1.zip › FIGURE5-source data1/FIGURE5J/Figure5J.anitkazrin.jpg]

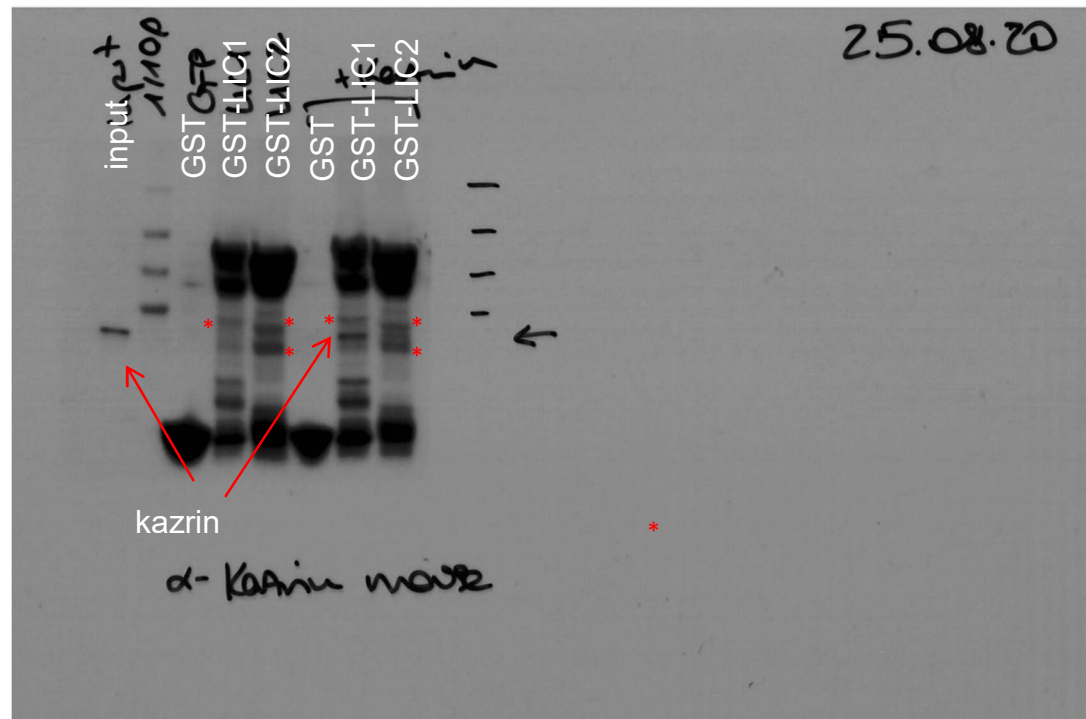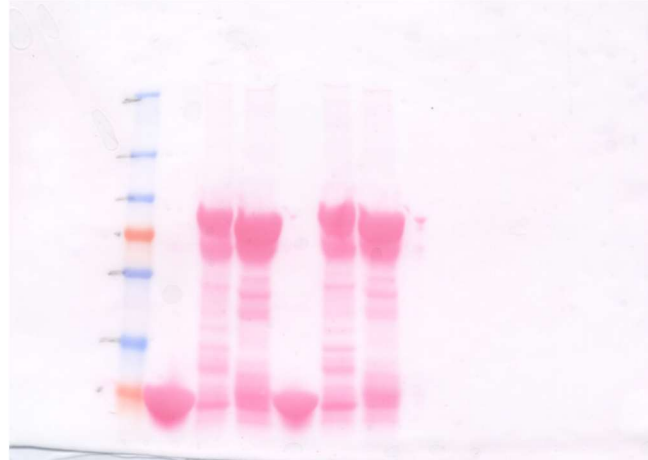

Supplement: Figure 5—source data 1. [file elife-83793-fig5-data1.zip › FIGURE5-source data1/FIGURE5J/FIGURE5J.pdf]

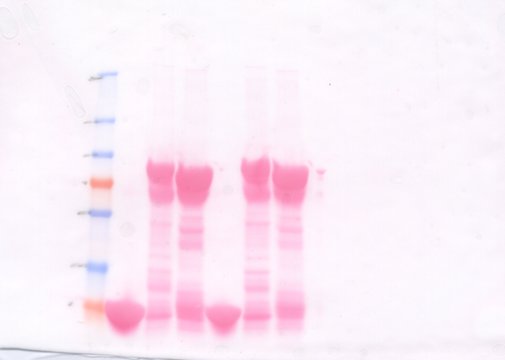

Supplement: Figure 5—source data 1. [file elife-83793-fig5-data1.zip › FIGURE5-source data1/FIGURE5J/Figure5J.ponceau.jpg]

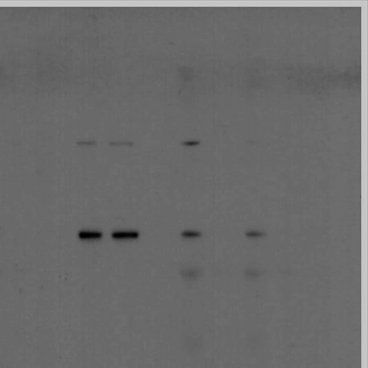

Supplement: Figure 5—source data 1. [file elife-83793-fig5-data1.zip › FIGURE5-source data1/FIGURE5K/figure 5K.antip150.anticortactin.jpg]

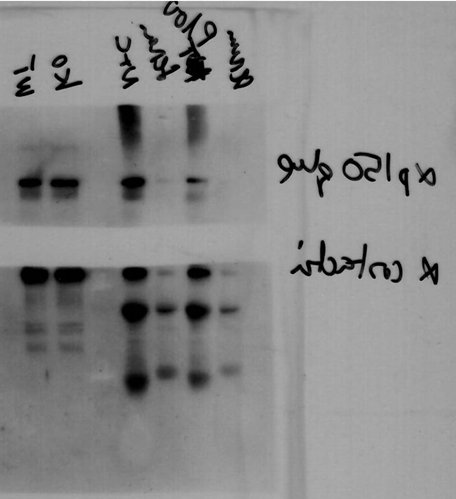

Supplement: Figure 5—source data 1. [file elife-83793-fig5-data1.zip › FIGURE5-source data1/FIGURE5K/figure 5K.antip150.anticortactin.long exposure.jpg]

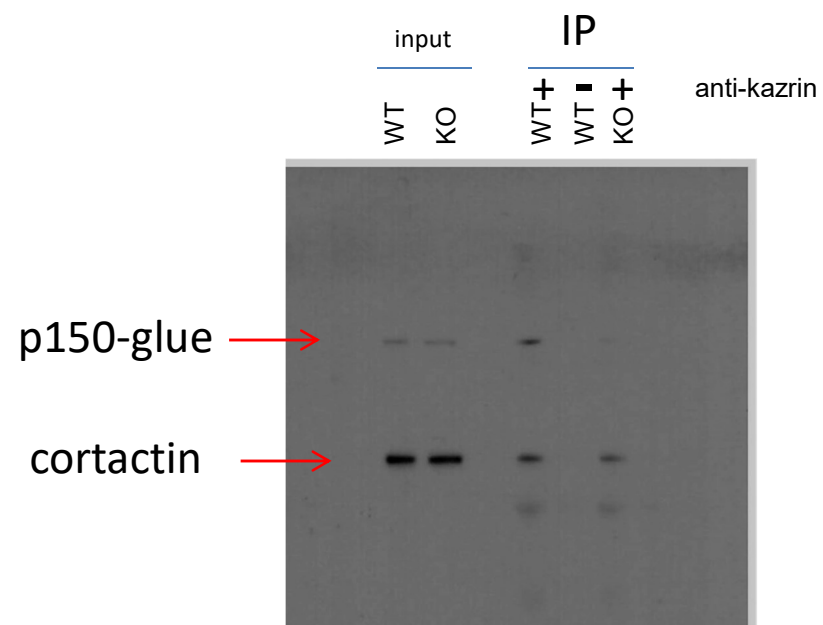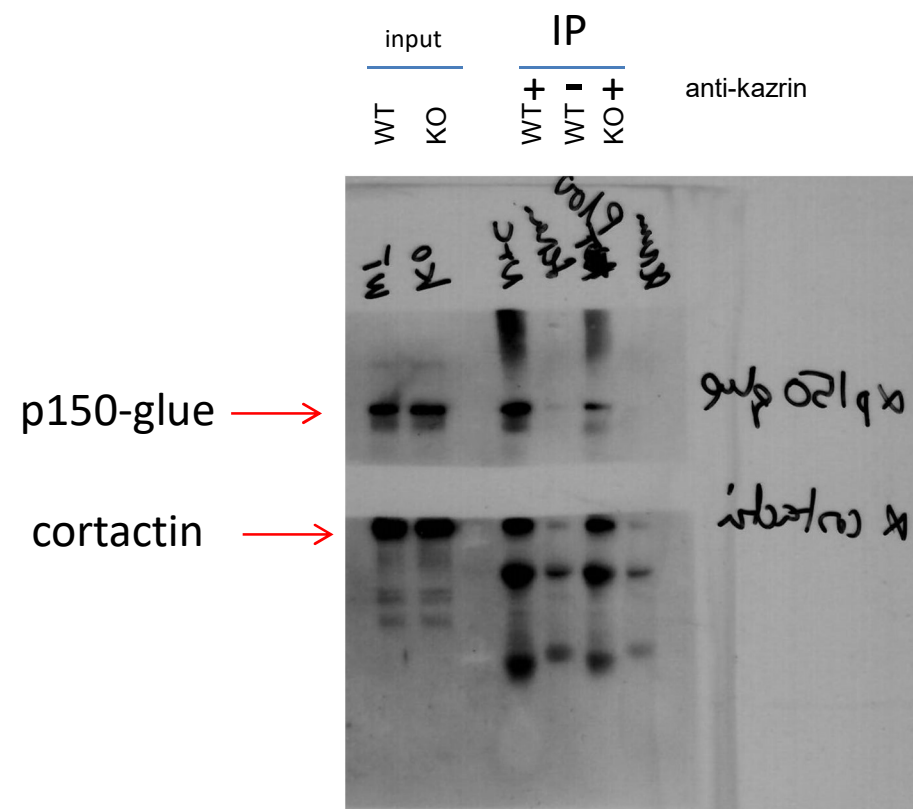

Long exposure

Supplement: Figure 5—source data 1. [file elife-83793-fig5-data1.zip › FIGURE5-source data1/FIGURE5K/FIGURE5K.pdf]
