# Supplementary material for: Kazrin promotes dynein/dynactin-dependent traffic from early to recycling endosomes
Source: eLife. 2023 Apr 25;12:e83793. doi: 10.7554/eLife.83793 (PMC10181827; doi:10.7554/eLife.83793)

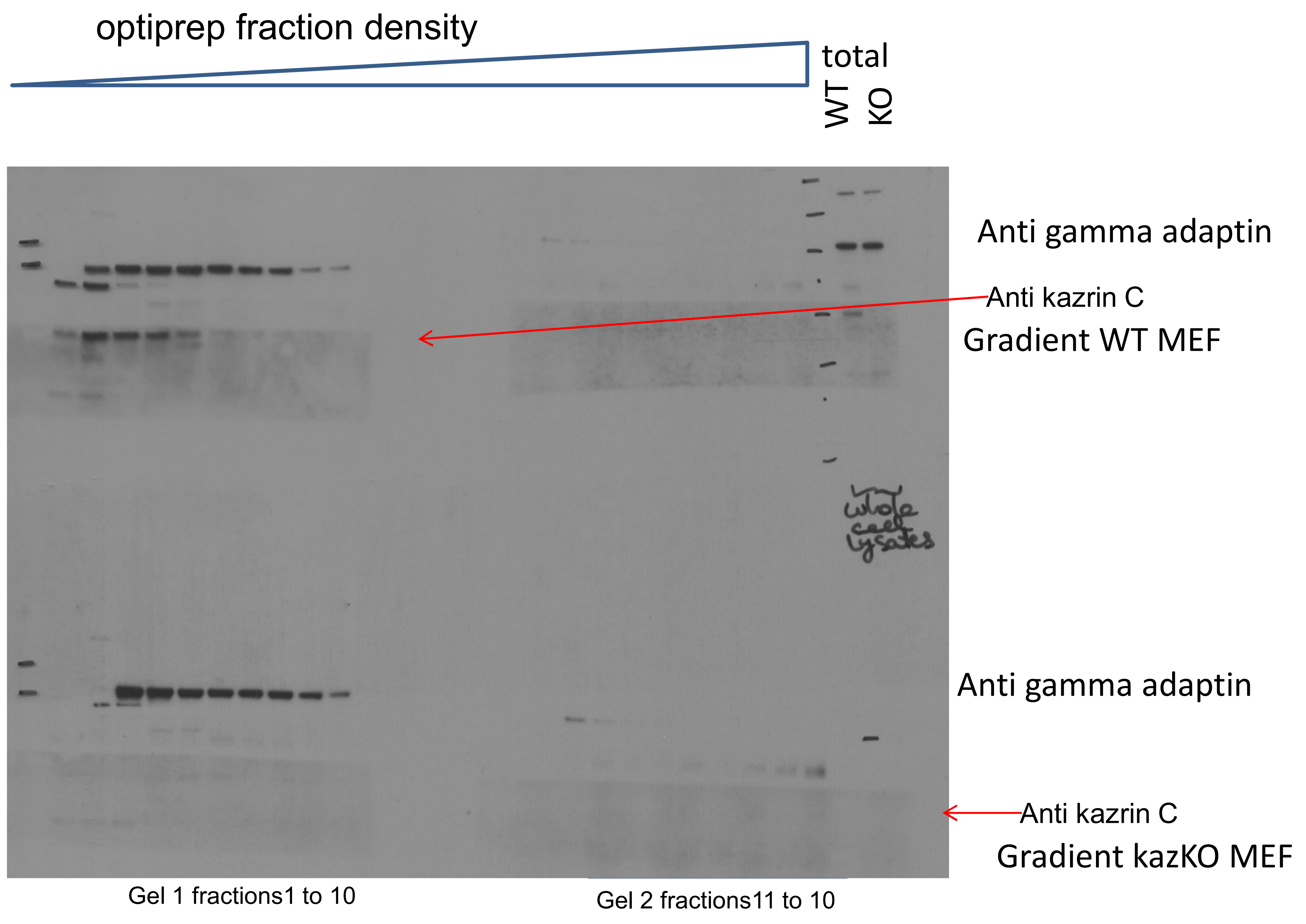

optiprep fraction density
total
WT
KO
Anti gamma adaptin
Anti kazrin C
Gradient WT MEF
whole cell lysates
Anti gamma adaptin
Anti kazrin C
Gradient kazKO MEF
Gel 1 fractions1 to 10
Gel 2 fractions11 to 10

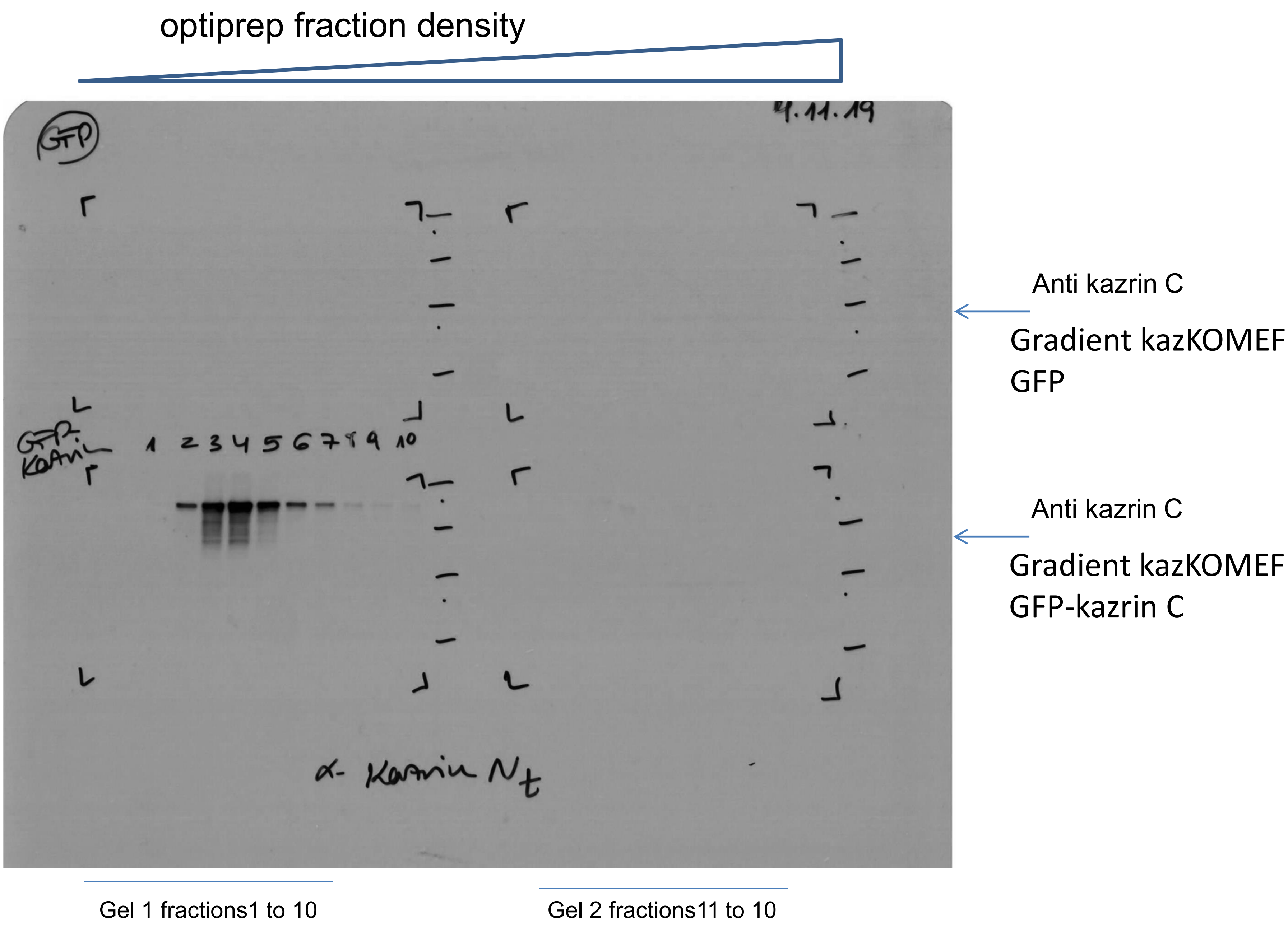
optiprep fraction density
GFP
4.11.19
GFP-Kazrin
1 2 3 4 5 6 7
9 10
α- Kazrin Nt
Anti kazrin C
Gradient kazKOMEF
GFP
Anti kazrin C
Gradient kazKOMEF
GFP-kazrin C
Gel 1 fractions1 to 10
Gel 2 fractions11 to 10

optiprep fraction density

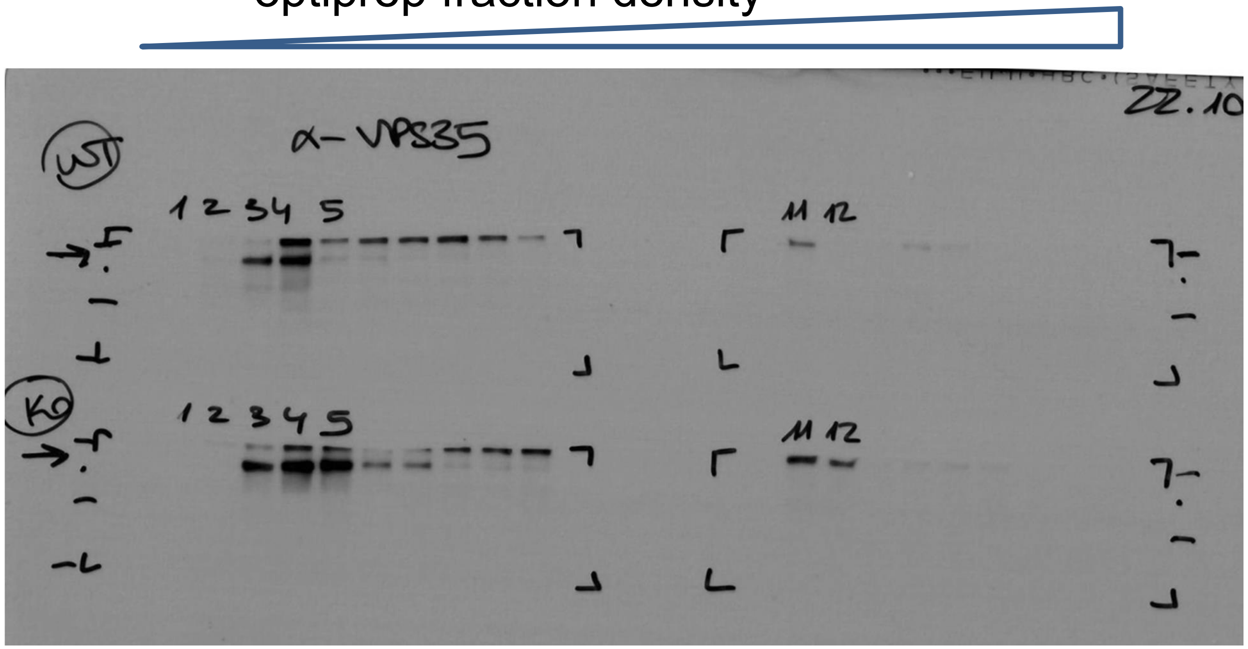

Anti Vps35

Gel 1 fractions1 to 10

Gel 2 fractions11 to 10

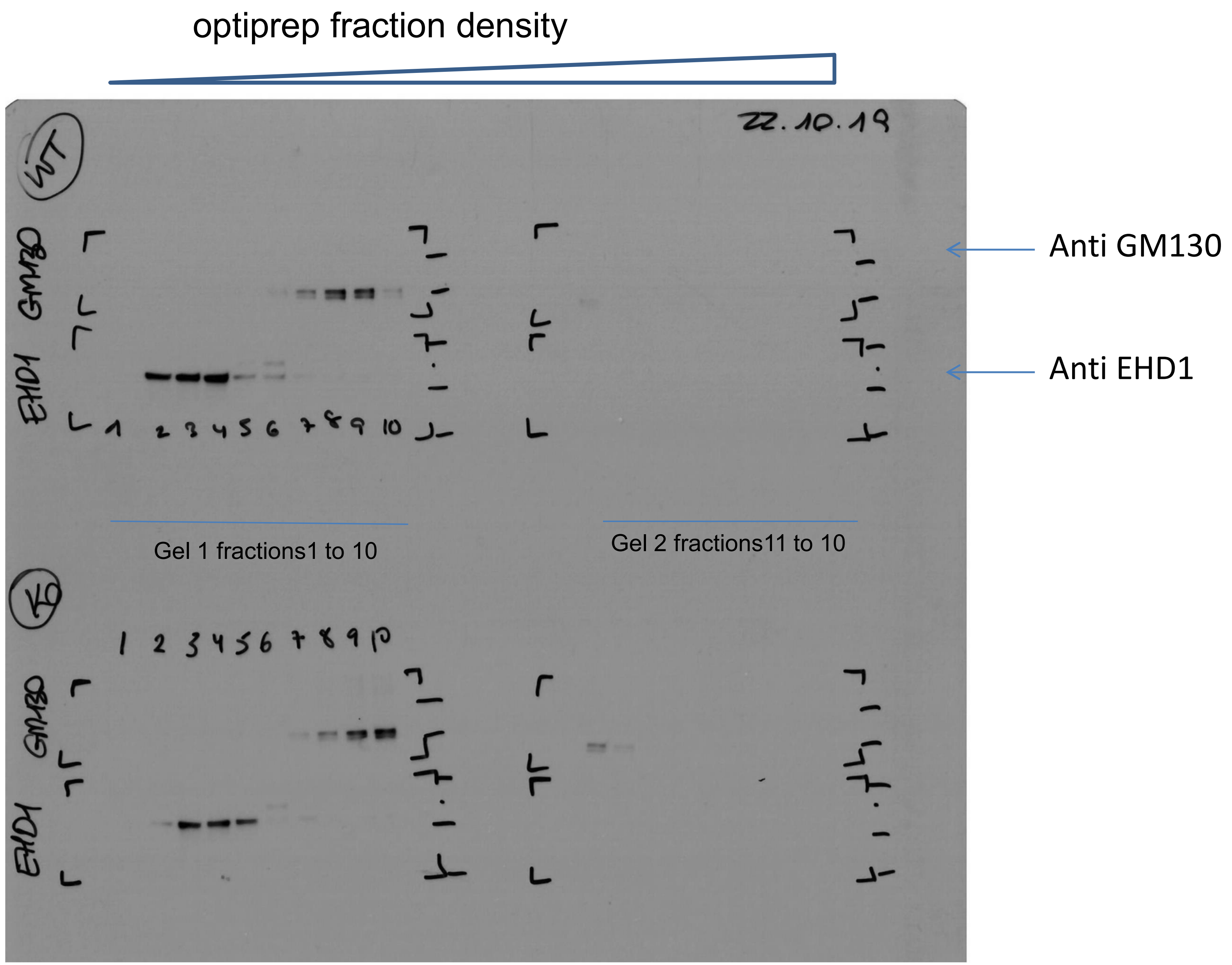
optiprep fraction density
22.10.19
WT
GM130
EHD1
1 2 3 4 5 6 7 8 9 10
Anti GM130
Anti EHD1
Gel 1 fractions1 to 10
Gel 2 fractions11 to 10
KO
1 2 3 4 5 6 7 8 9 10
GM130
EHD1

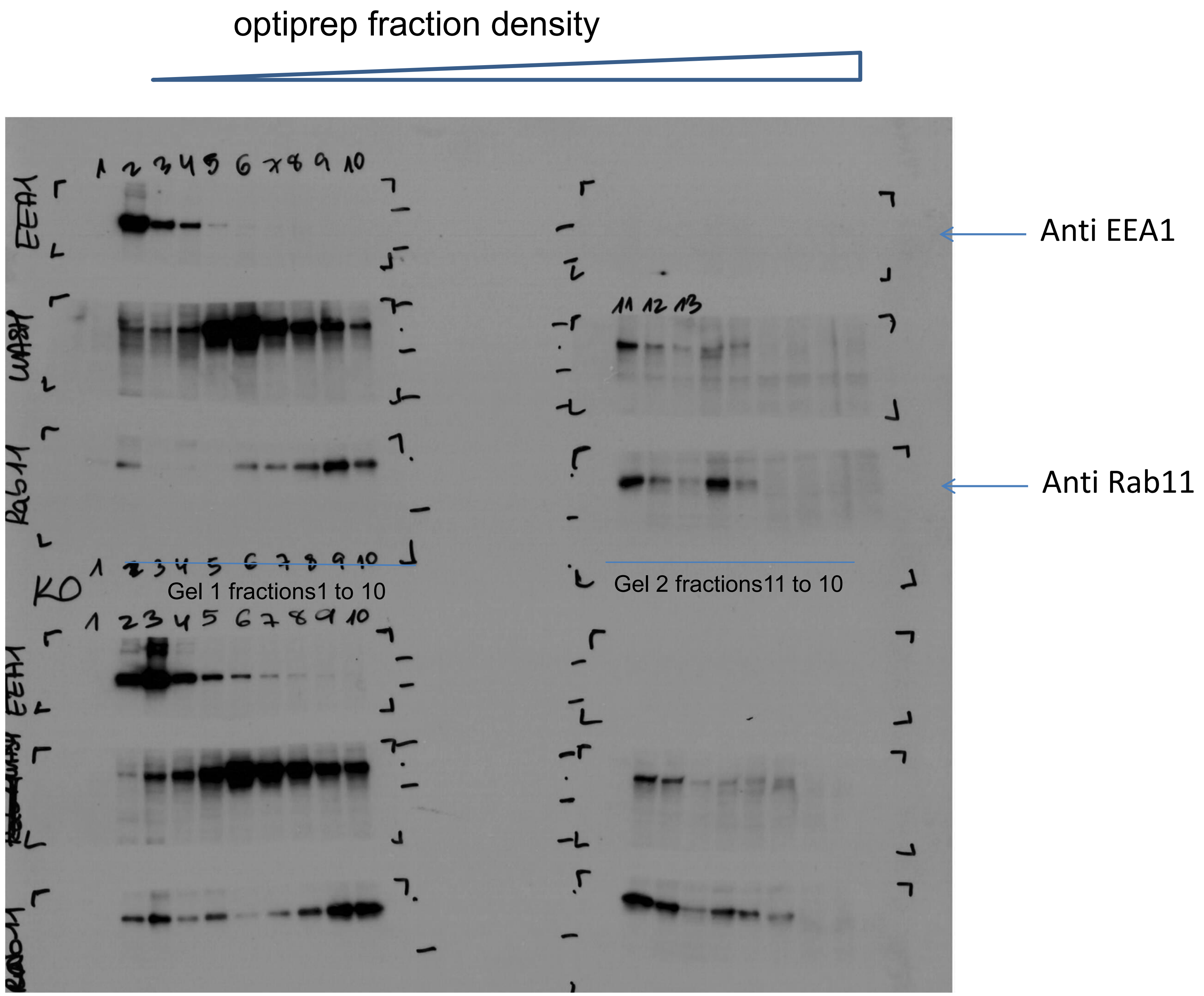

optiprep fraction density
Anti EEA1
Anti Rab11
Gel 1 fractions1 to 10
Gel 2 fractions11 to 10
KO

optiprep fraction density

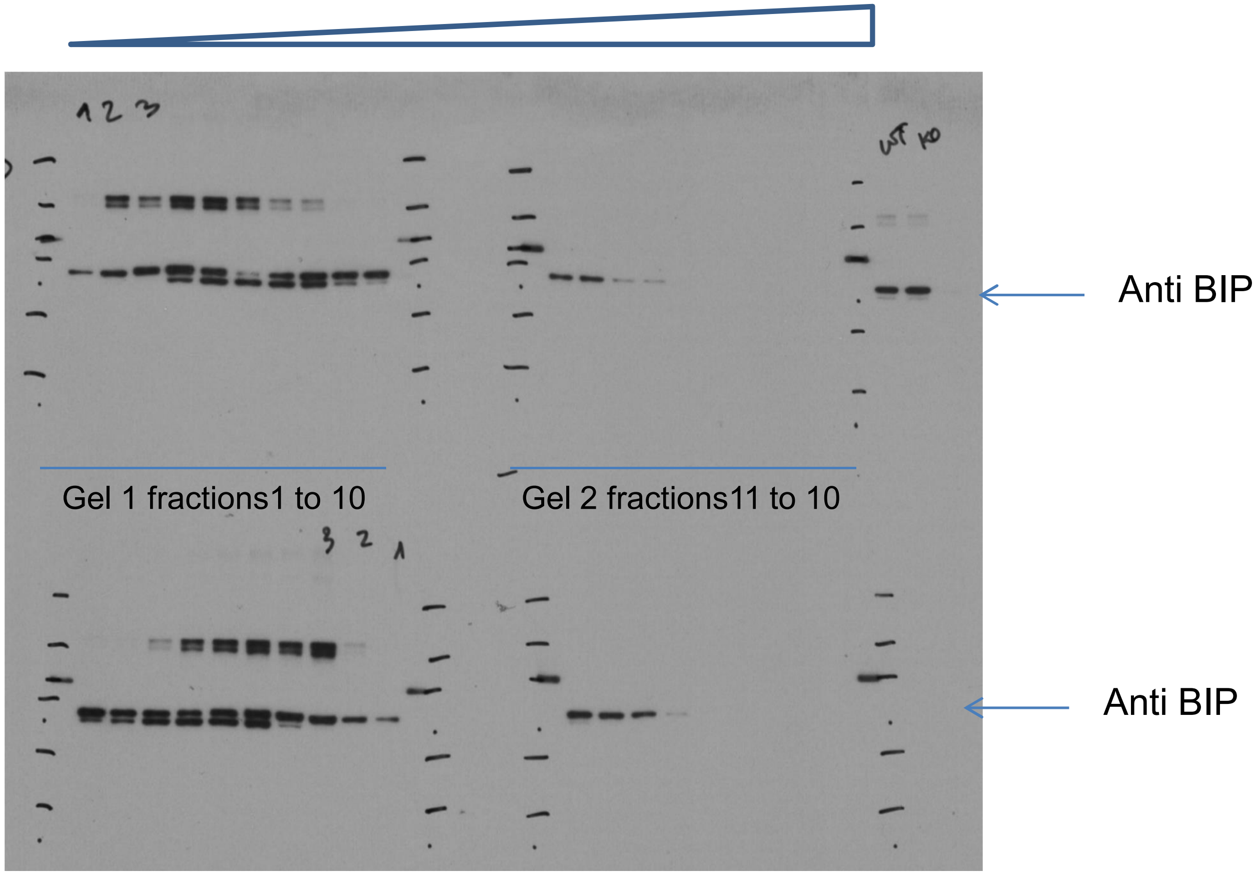

Supplement: Figure 3—source data 1. [file elife-83793-fig3-data1.zip › FIGURE3-source data/FIGURE3A/FIGURE3A.pdf]
