# Supplementary material for: Kazrin promotes dynein/dynactin-dependent traffic from early to recycling endosomes
Source: eLife. 2023 Apr 25;12:e83793. doi: 10.7554/eLife.83793 (PMC10181827; doi:10.7554/eLife.83793)
Supplement: Figure 3—source data 1. [file elife-83793-fig3-data1.zip › FIGURE3-source data/FIGURE3D/FIGURE3D.pdf]

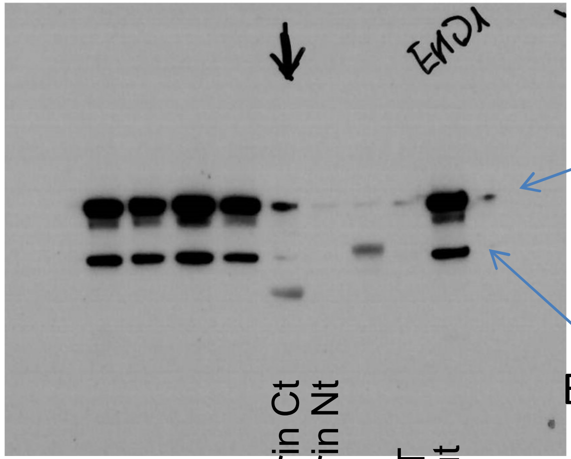

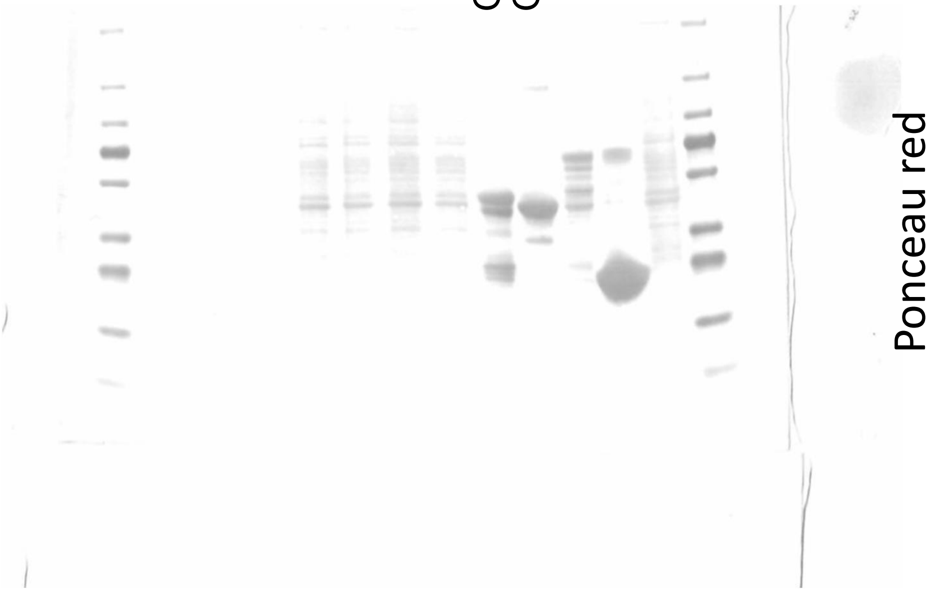

Anti EHD1 & anti-Gamma adaptin

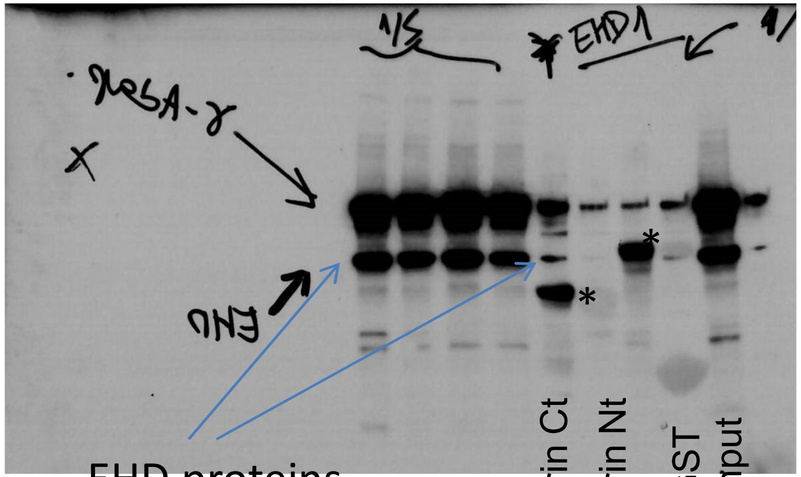

Anti EHD1 & anti-Gamma adaptin
Longer exposure

* GST constructs recognized by the secondary antibody
