# Supplementary material for: Kazrin promotes dynein/dynactin-dependent traffic from early to recycling endosomes
Source: eLife. 2023 Apr 25;12:e83793. doi: 10.7554/eLife.83793 (PMC10181827; doi:10.7554/eLife.83793)
Supplement: Figure 5—source data 1. [file elife-83793-fig5-data1.zip › FIGURE5-source data1/FIGURE5I/FIGURE5I.pdf]

Anti-p190-glued

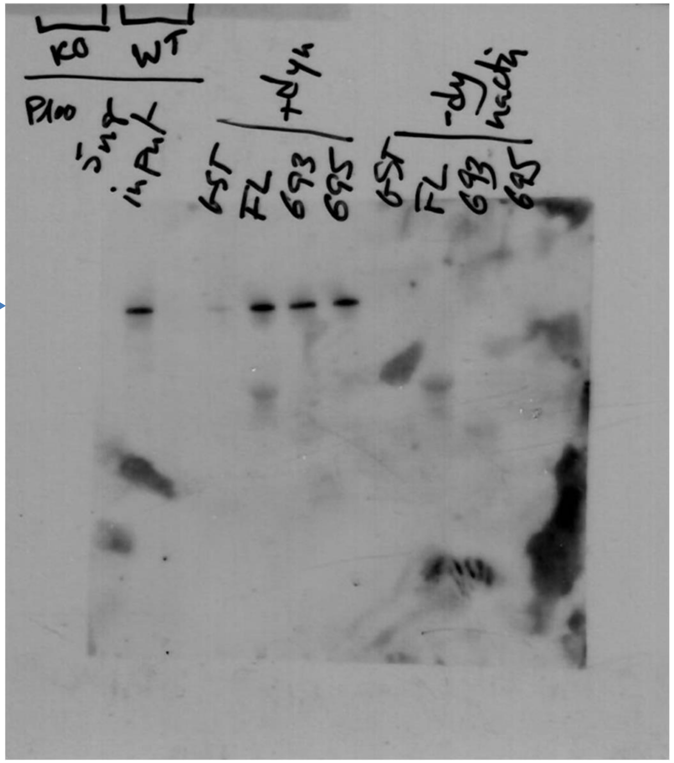

Input (5 ng)

GST

GST-Kaz C

GST-Kaz C Nt (aa1-176)

GST-Kaz C Ct (aa250-327)

GST

GST-Kaz C

GST-Kaz C Nt (aa1-176)

GST-Kaz C Ct (aa250-327)

Plus dynactin

Minus dynactin

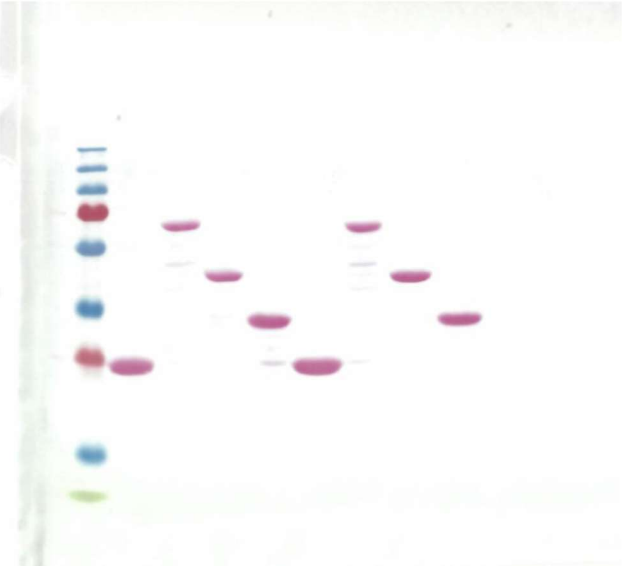

Input (5 ng)

GST

GST-Kaz C

GST-Kaz C Nt (aa1-176)

GST-Kaz C Ct (aa250-327)

GST

GST-Kaz C

GST-Kaz C Nt (aa1-176)

GST-Kaz C Ct (aa250-327)

Plus dynactin

Minus dynactin

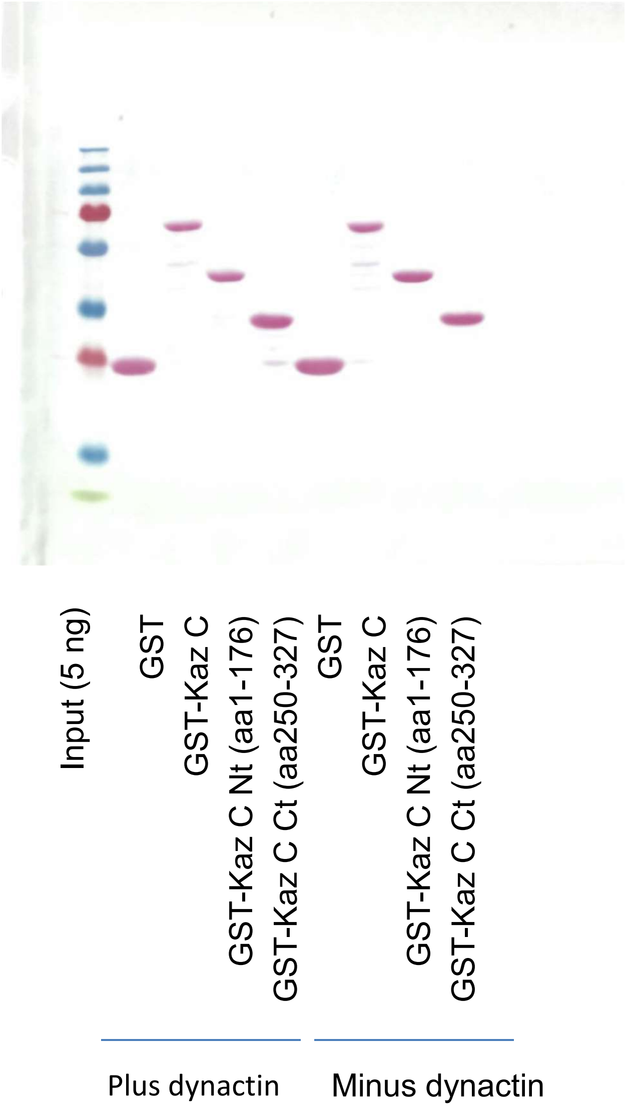
Input (5 ng)
GST
GST-Kaz C
GST-Kaz C Nt (aa1-176)
GST-Kaz C Ct (aa250-327)
GST
GST-Kaz C
GST-Kaz C Nt (aa1-176)
GST-Kaz C Ct (aa250-327)
Plus dynactin
Minus dynactin
