# Supplementary material for: Kazrin promotes dynein/dynactin-dependent traffic from early to recycling endosomes
Source: eLife. 2023 Apr 25;12:e83793. doi: 10.7554/eLife.83793 (PMC10181827; doi:10.7554/eLife.83793)
Supplement: Figure 1—figure supplement 2—source data 1. [file FIgure1-figure suplement2B.pdf › FIGURE1-figure supplement2-source data/FIgure1-figure suplement2B.pdf]

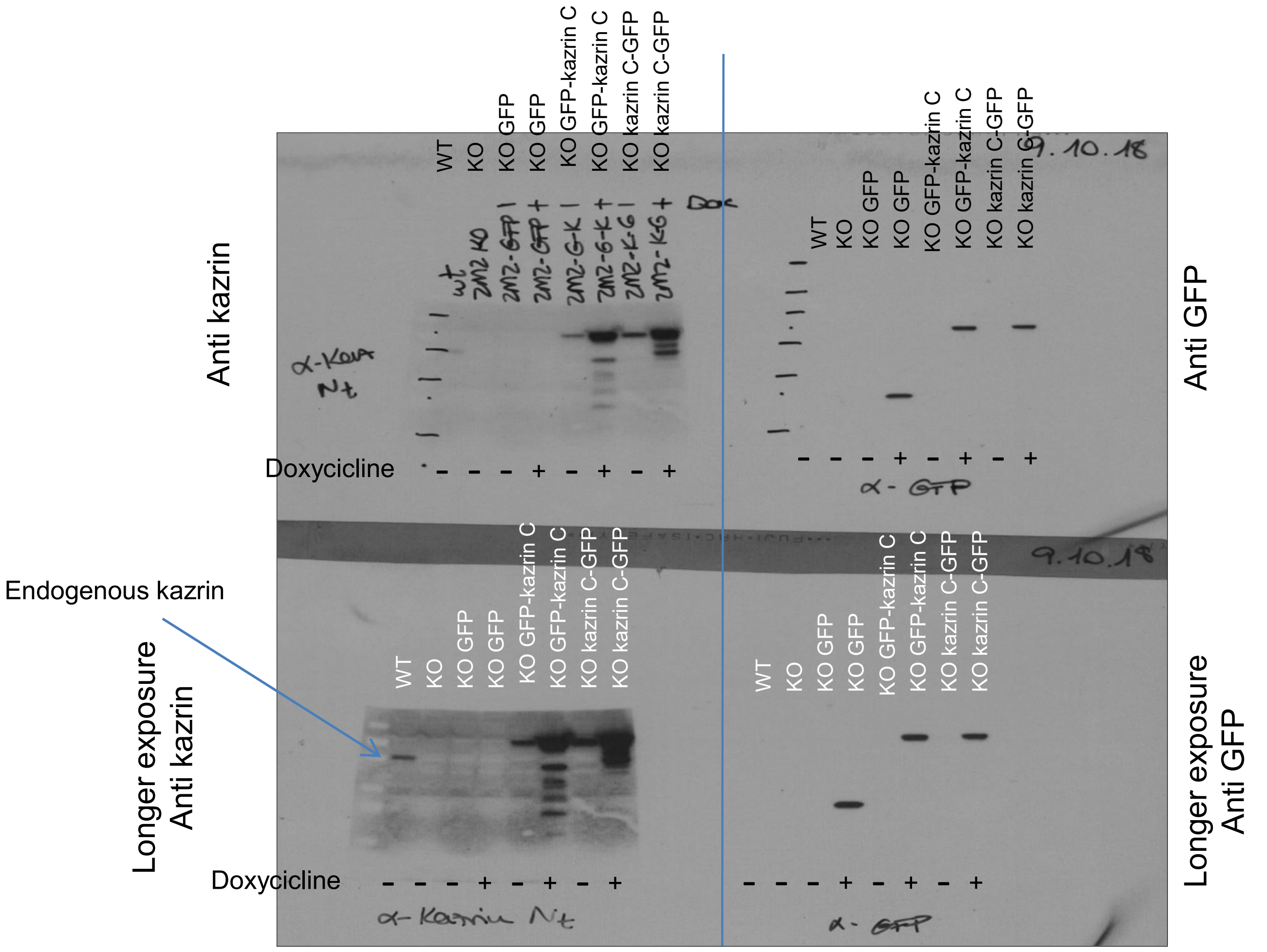
Anti kazrin
WT
KO
KO GFP
KO GFP
KO GFP-kazrin C
KO GFP-kazrin C
KO kazrin C-GFP
KO kazrin C-GFP
Doxycicline
- - - + - + - +
Anti GFP
WT
KO
KO GFP
KO GFP
KO GFP-kazrin C
KO GFP-kazrin C
KO kazrin C-GFP
KO kazrin C-GFP
- - - + - + - +
Endogenous kazrin
Longer exposure
Anti kazrin
WT
KO
KO GFP
KO GFP
KO GFP-kazrin C
KO GFP-kazrin C
KO kazrin C-GFP
KO kazrin C-GFP
Doxycicline
- - - + - + - +
Longer exposure
Anti GFP
WT
KO
KO GFP
KO GFP
KO GFP-kazrin C
KO GFP-kazrin C
KO kazrin C-GFP
KO kazrin C-GFP
- - - + - + - +
