# Supplementary material for: Kazrin promotes dynein/dynactin-dependent traffic from early to recycling endosomes
Source: eLife. 2023 Apr 25;12:e83793. doi: 10.7554/eLife.83793 (PMC10181827; doi:10.7554/eLife.83793)
Supplement: Figure 4—figure supplement 1—source data 1. [file FIGURE4-figure supplement1.pdf › FIGURE4-figure supplement1-source data/FIGURE4-figure supplement1.pdf]

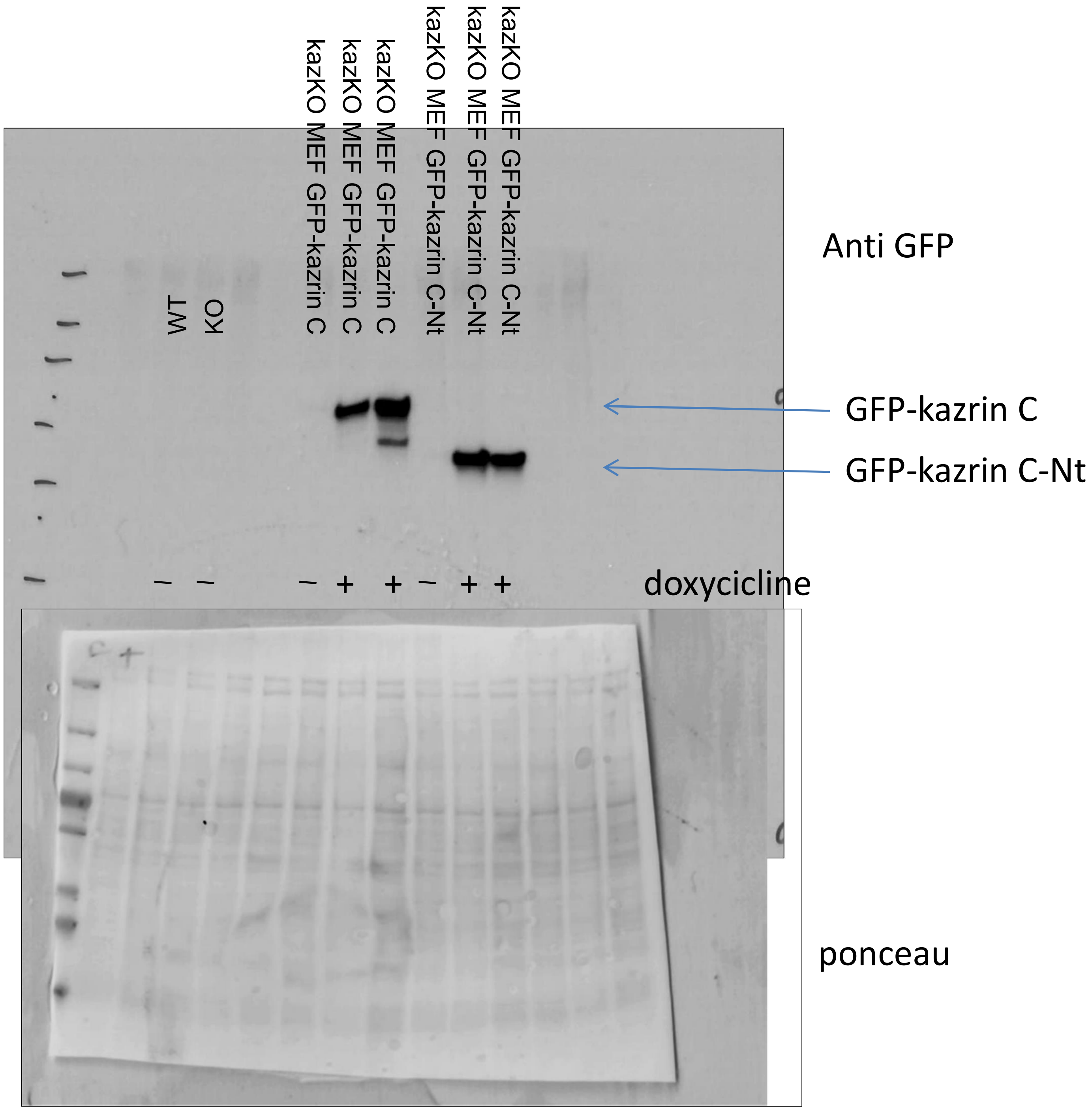
WT
KO
kazKO MEF GFP-kazrin C
kazKO MEF GFP-kazrin C
kazKO MEF GFP-kazrin C
kazKO MEF GFP-kazrin C-Nt
kazKO MEF GFP-kazrin C-Nt
kazKO MEF GFP-kazrin C-Nt
Anti GFP
GFP-kazrin C
GFP-kazrin C-Nt
– – – + + – + + doxycicline
ponceau
